# Identification of macrocyclic peptides which activate bacterial cylindrical proteases†

**DOI:** 10.1039/d3md00136a

**Published:** 2023-05-17

**Authors:** Raoul Walther, Linda M. Westermann, Sheiliza Carmali, Sophie E. Jackson, Heike Brötz-Oesterhelt, David R. Spring

**Affiliations:** a Yusuf Hamied Department of Chemistry, University of Cambridge Lensfield Road CB2 1EW Cambridge UK spring@ch.cam.ac.uk; b Interfaculty Institute of Microbiology and Infection Medicine, Dept. of Bioactive Compounds, University of Tübingen Auf der Morgenstelle 28 72076 Tübingen Germany heike.broetz-oesterhelt@uni-tuebingen.de; c School of Pharmacy, Queen's University Belfast BT9 7BL Belfast UK; d Cluster of Excellence Controlling Microbes to Fight Infections Germany

## Abstract

The caseinolytic protease complex ClpXP is an important house-keeping enzyme in prokaryotes charged with the removal and degradation of misfolded and aggregated proteins and performing regulatory proteolysis. Dysregulation of its function, particularly by inhibition or allosteric activation of the proteolytic core ClpP, has proven to be a promising strategy to reduce virulence and eradicate persistent bacterial infections. Here, we report a rational drug-design approach to identify macrocyclic peptides which increase proteolysis by ClpP. This work expands the understanding of ClpP dynamics and sheds light on the conformational control exerted by its binding partner, the chaperone ClpX, by means of a chemical approach. The identified macrocyclic peptide ligands may, in the future, serve as a starting point for the development of ClpP activators for antibacterial applications.

## Abstract 2

This work reports a divergent peptide stapling strategy to identify macrocyclic peptides which increase the proteolytic activity of the proteolytic core of the caseinolytic protease.

## Graphical abstract

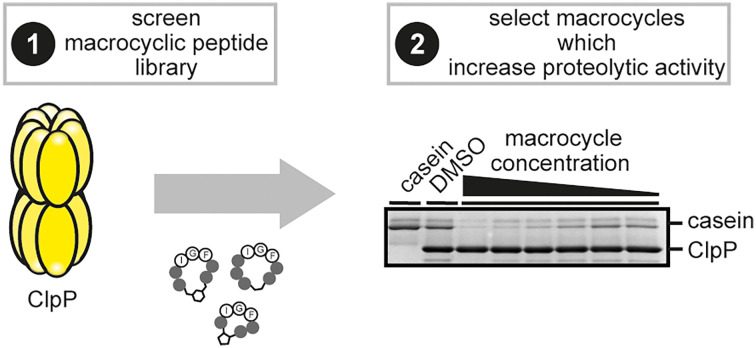

## Introduction

**Fig. 1:**
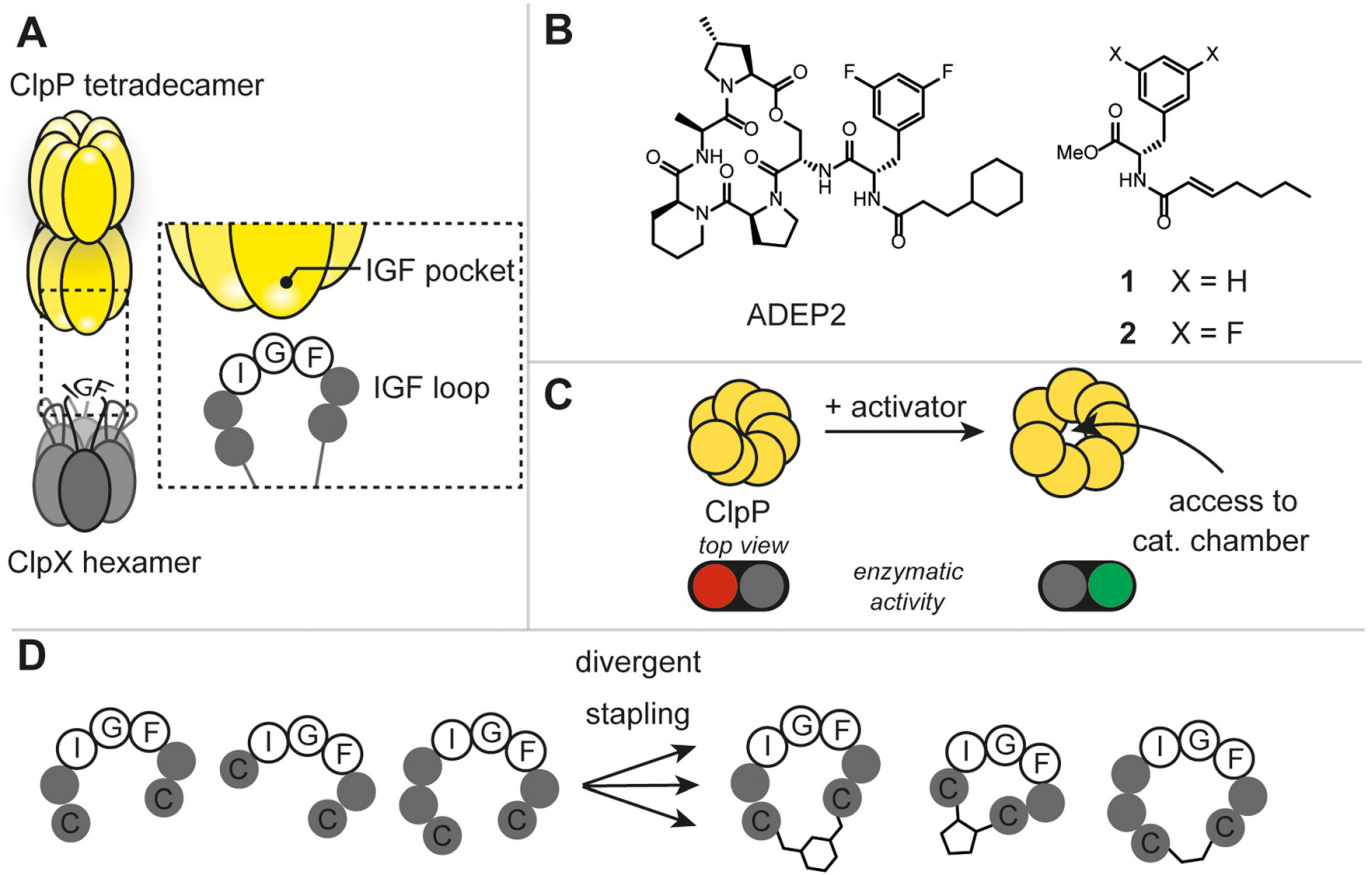
A) Schematic illustration of the caseinolytic protease complex consisting of the tetradecameric ClpP and the hexameric ATPase (ClpX) with the IGF-loops highlighted. B) Selected reported allosteric activators of the caseinolytic protease (ClpP). C) Schematic illustration of the opening of the apical pore upon activator binding. D) Diversity-oriented stapling approach varying the linear peptide sequence and two-component staple.

The caseinolytic protease complex (ClpXP), comprising the tetradecameric proteolytic core (ClpP) and a hexameric Clp-ATPase, ClpX, has been subject to intensive research due to its fundamental role in bacterial proteostasis, stress response and other regulatory processes (Fig. 1A).^1^ Despite this, rational target-selection programs in antibacterial discovery did not single out ClpXP as a promising target, presumably, because it is not essential for survival in the majority of prokaryotes.^1*c*^ It was not until the discovery of the natural product-derived acyldepsipeptide antibiotics (ADEP) that ClpXP became a promising antibacterial target (Fig. 1B).^2^ Mode of action studies revealed that ADEP binds tightly to ClpP and inhibits its interaction with ClpX. Intriguingly, as opposed to most antibiotics used in the clinic, ADEP is not an inhibitor of enzymatic function, but instead allosterically increases enzymatic activity of ClpP and causes uncontrolled protein degradation (Fig. 1C). Mutational analyses have shown that the essential binding epitope of ClpX to ClpP is the tripeptide ‘I/LGF’.^3^ Prior to high resolution cryo-EM structures of ClpXP, ADEP served as a tool mimicking the binding of the ClpX tripeptide ‘IGF’ and shed light on the induced dynamics and structural consequences on ClpP.^1*a*,*b*^ To date, ClpX-induced structural and mechanistic consequences continue to be a debated topic. For instance, in the three ClpXP cryo-EM studies published to date, substantial differences in axial pore diameter were observed.^4^ Thus, we envisaged that increasing the arsenal of more ‘ClpX-like’ ligands could contribute to the discussion and clarify some of the unknowns. This work aims at answering 1) whether the rational design of peptidomimetic macrocycles bearing the IGF binding epitope through a two-component stapling approach is feasible, 2) whether a focused library can deliver ligands against ClpP, and 3) whether binding results in any regulatory control of the protease.

## Results and discussion

Due to the lack of prior known peptide-based low molecular weight ligands to ClpP (other than ADEP), we opted for a diversity-oriented stapling strategy^5^ varying the linear peptide sequence and the two-component staples for macrocyclization (Fig. 1D). For macrocyclization, we selected a two-component peptide stapling strategy.^6^ Specifically, we used a cysteine bis-alkylation method,^7^ due to its straightforward synthetic implementation and potential for further chemical functionalization, such as fluorescent tags for microscopy or cell-penetrating tags.^8^ Considering the structural knowledge available, we kept the evolutionary conserved tripeptide binding epitope ‘IGF’ of ClpX constant and varied the number of flanking amino acids based on the natural epitope from *Escherichia coli* (*E. coli*).

**Fig. 2:**
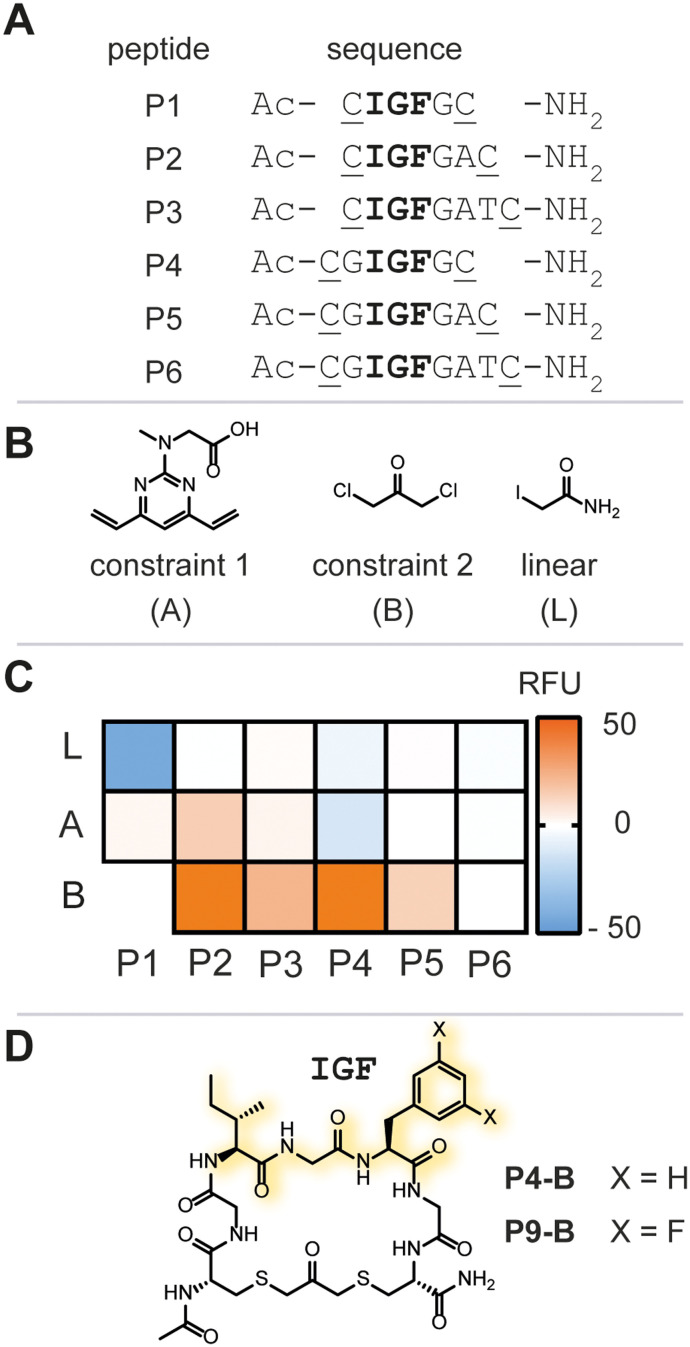
A) Summary of linear peptide sequences. The IGF epitope is highlighted in bold and positions for cysteine side chain macrocyclization are underlined. B) Utilized two-component staples A and B, and iodoacetamide to generate linear peptide controls L. C) Heat-map of the biochemical hit identification. Reagents and conditions: 500 nM Ec.ClpP, 7 μM beta-casein/FITC-casein (70/30), 200 μM peptide library after 24 h at 37 °C. RFU = relative fluorescence units. D) Chemical structure of **P4-B** and **P9-B**.

As a starting point, we chose six peptide sequences (**P1**–**P6**, Fig. 2A) bearing C- and N-terminal cysteine residues for ring closure *via* alkylation. Linear peptide precursors **P1**–**P6** were prepared by conventional Fmoc-based solid-phase peptide synthesis (see ESI† for detailed experimental protocols). Peptides were synthesized using rink-amide resin resulting in C-terminal amides and the N-terminus was capped with acetic anhydride. The staples were varied with respect to introduced carbon chain length (three, five, and seven) to explore the impact of macrocycle size and rigidity on binding, namely 1,3 dichloroacetone (**B**),^7*b*^ 1,3-bis(bromomethyl)benzene,‡‡Initial stapling attempts resulted in poor aqueous solubility of the resulting macrocycles and concomitant difficulties during purification. Thus, this sequence of macrocycles was abandoned.^7*c*^ and 4,6-divinylpyrimidine (**A**),^7*a*^ respectively (Fig. 2B, see ESI† for detailed experimental protocols).

Next, we screened for enzymatic activation of ClpP to quickly assess the chemical library for binding and understand whether IGF-bearing macrocyclic peptides could modulate ClpP enzymatic function. Modulation of ClpP was assessed using a biochemical assay monitoring the enzymatic function of the protease based on previously reported successes for the identification of allosteric activators of ClpP.^9^ In the absence of an allosteric ligand, such as ADEP, the apical pore of ClpP is constricted, only allowing small peptides to enter the catalytic chamber. In contrast, ADEP binding to the apical hydrophobic pocket results in apical pore opening to a diameter of ∼20 Å, allowing access of unstructured proteins to the catalytic chamber of the protease (Fig. 1C). FITC-casein, a protein labeled with fluorescein to the extent of self-quenching, is a suitable substrate for this assay, as it can only enter the proteolytic core upon apical pore opening and is indirectly a measure for the conformational state of the protease.^2,10^ Enzymatic degradation of FITC-casein alleviates self-quenching giving rise to an increased fluorescence intensity and an easy readout for our purpose to identify any ligand that positively modulates the enzymatic activity of recombinant *E. coli* ClpP (Ec.ClpP).

Ec.ClpP was incubated for 24 h at 37 °C in the presence of a peptide library, or vehicle (DMSO) and fluorescence intensity was measured thereafter (Fig. 2C and S1†). All linear peptide controls (**P1**–**P6-L**) showed no increase in fluorescence over the negative control. The macrocycles based on stapling with **A** showed only marginal increase in fluorescence and this indicates that a 7-atom staple is too large. Constriction of the linear peptide sequences with the smaller staple B showed an impact on the overall fluorescence intensity, with **P2-B** and **P4-B** (Fig. 2D) demonstrating a significant increase of fluorescence intensity over the DMSO-treated sample. This was reproducibly confirmed in three independent experiments (varying the batch of enzyme). Further evaluation of **P2-B** revealed that the presence or absence of Ec.ClpP did not significantly change the fluorescence signal and that the basal fluorescence response was significantly higher than the DMSO control, rendering it a false positive hit (Fig. S2†). Fortunately, **P4-B** did not exhibit this behaviour and was used in all further experiments. The initial focused library screen illustrated that peptide linear sequence, chemical constraint, and macrocycle size play an intricate role in selecting an IGF conformation which activates Ec.ClpP function.

**Fig. 3:**
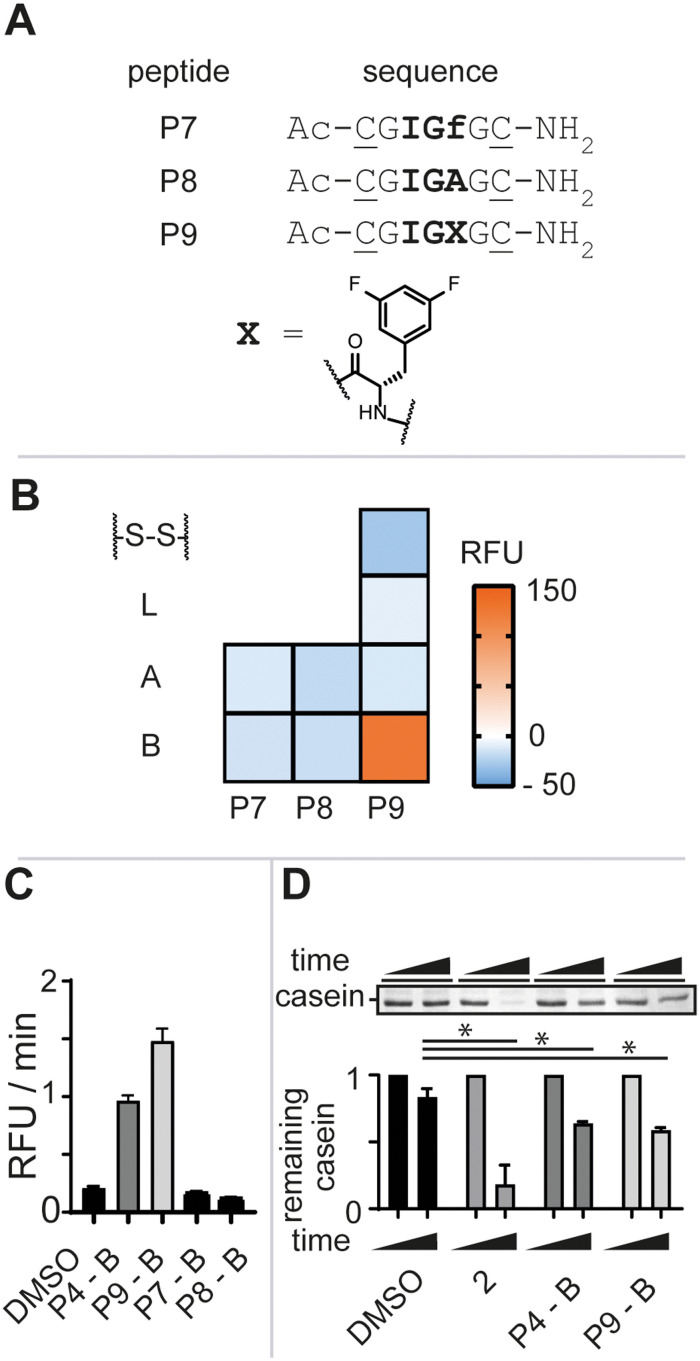
A) Linear peptide sequences. The tripeptide sequence is highlighted in bold and positions for cysteine side-chain macrocyclization are underlined. B) Heat-map of the biochemical hit identification. Reagents and conditions: 500 nM Ec.ClpP, 7 μM beta-casein/FITC-casein (70/30), 200 μM peptide library after 24 h at 37 °C. RFU = relative fluorescence units. C) Rate comparison of the digestion of FITC-casein by the investigated peptide ligands at 200 μM. D) SDS-PAGE analysis of Ec.ClpP-dependent beta-casein digestion by the investigated peptide ligands and 2 at 200 μM after 24 h and densitometric analysis thereof. Data are represented as mean ± SD of at least three independent experiments. Statistical significance was evaluated using an un-paired two-tailed *t*-test.

To further gauge the sequence specificity of the tripeptide epitope IGF and rule out unspecific binding, we synthesized peptides **P7** and **P8** substituting l-phenylalanine for its enantiomer d-phenylalanine (f) and l-alanine (A), respectively (Fig. 3A, see ESI† for detailed experimental protocols). Again, the isolated macrocycles were subjected to our enzymatic assay and the negative control peptide macrocycles showed no stimulation of enzymatic activity (Fig. 3B). The requirement of the natural configuration and sequence of the IGF loop and of the ADEP Phe linker for activation supported the notion that the increase in fluorescence was a direct consequence of **P4-B** binding to ClpP and inducing a conformational change reminiscent of ADEP^2^ and other activators.^9*b*,11^

ADEP structure–activity studies revealed that the introduction of two fluoro-substituents on the phenylalanine residue markedly increased the bioactivity against *Staphylococcus aureus* (*S. aureus*).^12^ We thus opted to synthesize bis-fluorinated macrocyclic analogue to improve the activity of the lead macrocycle (see ESI† for detailed experimental protocols). Enzymatic activity was not observed for any of the synthesized analogues (**P9-A**, **P9-S-S**, or **P9-L**) other than the macrocycle with constraint B, **P9-B**, in agreement with our previous studies (Fig. 3B). **P9-B** exhibited a 1.5-fold improved activity over **P4-B** at equimolar concentrations, whereas the control macrocyclic peptides **P7-B** and **P8-B** were devoid of activity (Fig. 3C). Further validation of the **P4/9-B**-dependent control of Ec.ClpP function was confirmed by SDS-PAGE analysis of beta-casein digestion (Fig. 3D).§§A positive control 2 (ref. 15) (Fig. 1B) was included in the assay.

Given the high sequence conservation of the tripeptide epitope of ClpX across bacterial phyla,^3^ we were interested in understanding whether **P4-B** and **P9-B** could activate other homologues of ClpP. To this end, we expressed and isolated *S. aureus* ClpP (Sa.ClpP), one of the most relevant pathogens where ClpP activators have shown clinical potential.^2,13^

**Fig. 4:**
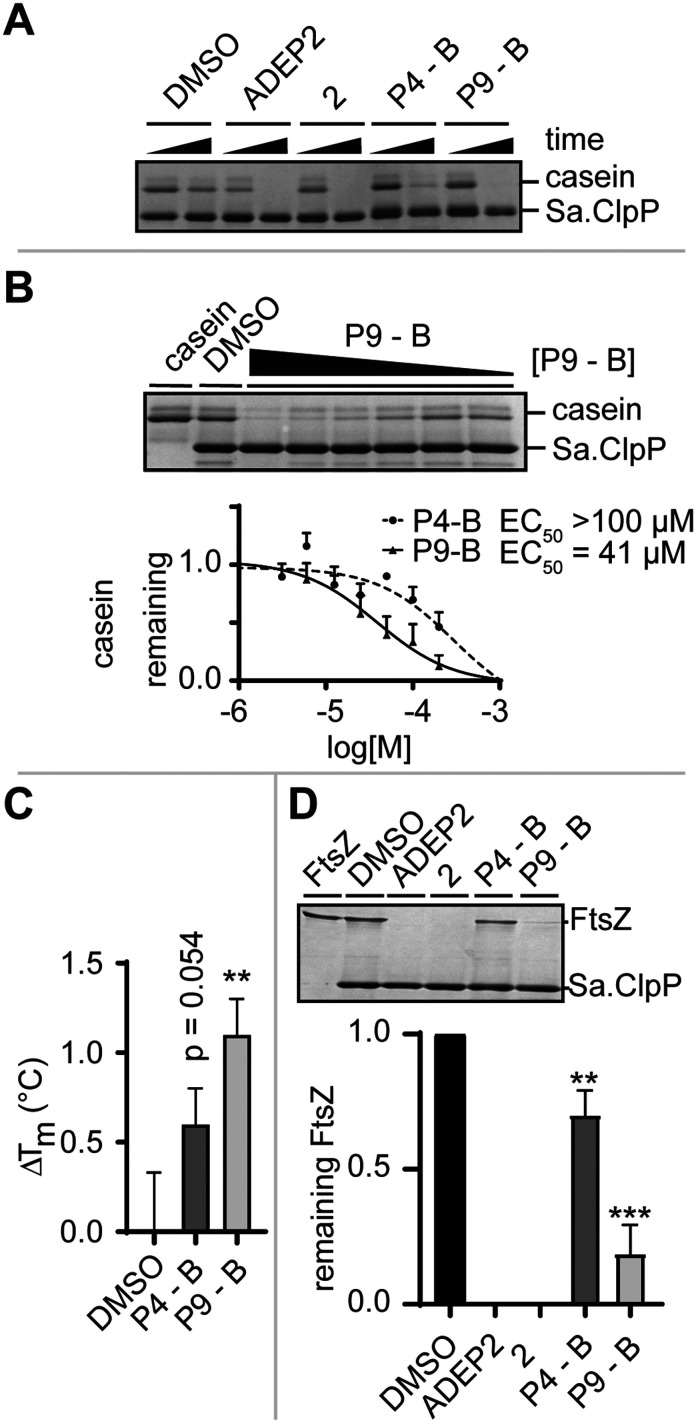
A) SDS-PAGE analysis of Sa.ClpP-dependent beta-casein digestion induced by allosteric activators (at 200 μM and ADEP2 at 10 μM final concentration after 24 h). B) Dose–response evaluation of **P4-B**- and **P9-B**-induced Sa.ClpP-dependent digestion of beta-casein. C) Change in melting temperature of Sa.ClpP in the presence or absence of **P4-B** and **P9-B** as determined by differential scanning fluorimetry. D) SDS-PAGE analysis of Sa.ClpP-dependent Sa.FtsZ digestion induced by allosteric activators (at 200 μM and ADEP2 at 10 μM final concentration after 24 h) and densitometric analysis thereof. Data represented as mean ± SD of at least three independent experiments. Statistical significance was evaluated using an un-paired two-tailed *t*-test.

We monitored the digestion of beta-casein by Sa.ClpP in the presence of ADEP2, 2 (Fig. 1B), **P4-B**, and **P9-B** (Fig. 2D). **P4-B** and **P9-B** were able to activate the enzymatic function of Sa.ClpP, indicating binding to other ClpP homologues (Fig. 4A). This observation was dose-dependent with an observed effective dose (EC_50_) of 41 μM for **P9-B** and >100 μM for **P4-B** (Fig. 4B). Binding was further validated by differential scanning fluorimetry and a mild stabilization of Sa.ClpP was observed in presence of **P4-B** and **P9-B** with an apparent increase in melting temperature of 0.6 ± 0.2 and 1.1 ± 0.2 °C, respectively (Fig. 4C and S3†).

Prior to high-resolution cryo-EM structures of ClpXP, much of the ClpX induced allostery has been deduced from ADEP binding to ClpP. Key structural features induced by ADEP are the observed axial pore opening and the selection for the extended state of ClpP aligning the catalytic triad in its active geometry. Additionally, ADEP assembles oligomeric forms of ClpP to its active tetradecamer. The enzymatic digestion experiments strongly suggest that the identified macrocycles induce proteolysis in a concentration-dependent manner. Now, we set out to answer whether **P4/9-B** are able to stabilize the tetradecameric form of Sa.ClpP. We artificially destabilized tetradecameric Sa.ClpP by changing the buffer from a compatible HEPES (pH 7.0) to an inactivating Tris-HCl buffer (pH 7.6). We speculate that the destabilization is due to a change in pH.^14^ The oligomeric state was monitored by gel-filtration experiments. ADEP2 and 2 (ref. 15) were able to retain the tetradecamer in Tris-HCl, unlike **P4/9-B** (Fig. S4†). This experiment demonstrates that ADEP-based high affinity ligands are able to stabilize oligomeric state of ClpP, whereas our ‘ClpX’-like ligands did not show the same stabilization, presumably due to their lower intrinsic affinity¶¶Affinity refers in this context to the estimated EC_50_ for casein degradation not to the binding constant. and require much higher concentrations (due to solubility issues of the macrocycles, concentrations exceeding 0.2 mM were not attempted). ClpX overcomes this obstacle by means of avidity and ClpX mutants lacking one or two ‘IGF’-loops show compromised association and dissociation kinetics to ClpP.^16^

Lastly, we set out to answer to what extent ADEP and other allosteric activators of ClpP resemble the activation process of ClpX. To this end, we set up a biochemical assay and monitored the digestion of FtsZ, an essential protein for cell division, which is the major determinant for the filamentation phenotype induced by ADEP-activated ClpP.^17^ Similarly to the digestion of beta-casein, ADEP2 and 2-treated samples activated Sa.ClpP, and FtsZ was digested. Incubation of **P4-B** and **P9-B** with ClpP also induced the digestion of FtsZ, yet less efficiently (Fig. 4D). This experiment is in agreement with the enzymatic digestion experiment of beta-casein and suggests that the structural and dynamic consequences of our IGF epitopes on ClpP are not substrate dependent, but are a result of the presence of the identified macrocycle, and strongly support a mechanism of axial pore opening. We performed docking studies of the identified macrocycles into the same pocket where ADEP binds (see ESI† for full details). **P9-B** showed an improved docking score over **P4-B**, in agreement with our experimental data (Table S4†).

The identified macrocyclic peptides require higher concentrations and longer incubation times than the high affinity ligands ADEP2 and 2. Nevertheless, the experiments illustrate that mimicking the IGF-loops of ClpX without its controlling elements results in proteolytically active ClpP and this holds promise for the development of macrocyclic peptide activators of ClpP. Of note, at this stage we cannot rule out that our identified macrocycles bind at another site than the suggested allosteric site of the IGF binding loop of ClpX. There is precedence where dipeptides and the protease inhibitor bortezomib are able to increase enzymatic function of ClpP homologues through another mechanism.^11^

## Conclusions

In summary, we succeeded in identifying macrocyclic peptide ligands against ClpP through a diversity-oriented stapling approach. Our biochemical analysis strongly suggests that the identified lead macrocyclic peptides (**P4-B** and **P9-B**) containing the IGF binding epitope induce enzymatic activity. The current study is the first report on successfully targeting the ClpP hydrophobic pocket by a synthetic macrocyclic peptide and lays a foundation for the rational design of ‘ClpX-like’ macrocyclic peptide activators of ClpP.

## Author contributions

Project was conceptualized by RW. Experiments were designed and performed by RW. Sa.ClpP was isolated by RW and LMW. RW and SC designed docking experiment, SC performed and interpreted data. SEJ, HBO and DRS supervised the project. All authors contributed to data interpretation and manuscript writing.

## Conflicts of interest

There are no conflicts to declare.

## Supplementary Material

MD-014-D3MD00136A-s001
