# Supplementary material for: Identification of macrocyclic peptides which activate bacterial cylindrical proteases
Source: RSC Med Chem. 2023 May 17;14(6):1186–91. doi: 10.1039/d3md00136a (PMC10285738; doi:10.1039/d3md00136a)
Supplement: MD-014-D3MD00136A-s001 [file MD-014-D3MD00136A-s001.pdf]

Electronic Supplementary Material (ESI) for RSC Medicinal Chemistry.
This journal is © The Royal Society of Chemistry 2023

Identification of macrocyclic peptides which activate bacterial cylindrical proteases

Raoul Walther[a], Linda M. Westermann[b], Sheiliza Carmali[c], Sophie E. Jackson[a], Heike Brötz-Oesterhelt*[b, d], David R. Spring*[a]

[a] Yusuf Hamied Department of Chemistry
University of Cambridge
Lensfield Road, CB2 1EW Cambridge, United Kingdom
E-mail: spring@ch.cam.ac.uk

[b] Interfaculty Institute of Microbiology and Infection Medicine, Dept. of Bioactive Compounds,
University of Tübingen
Auf der Morgenstelle 28, 72076 Tübingen, Germany
E-mail: heik.boetz-oesterhelt@uni-tuebingen.de

[c] School of Pharmacy, Queen's University Belfast, BT9 7BL Belfast, United Kingdom

[d] Cluster of Excellence Controlling Microbes to Fight Infections

Supporting Information

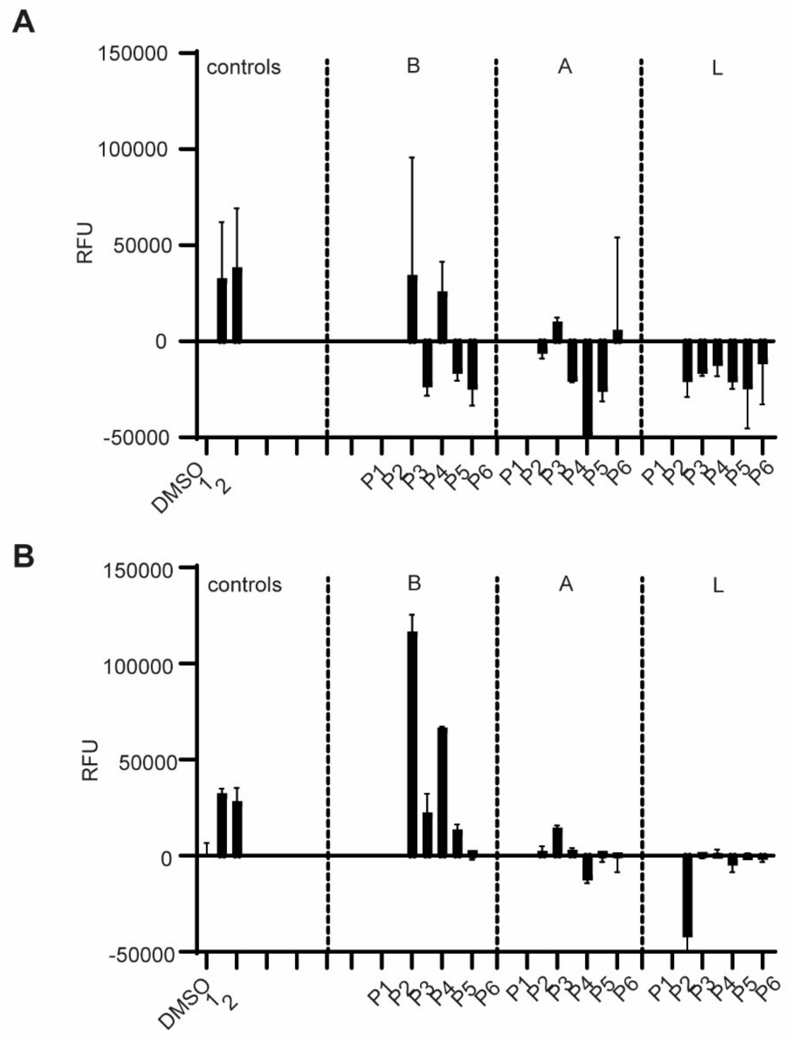

Supplementary Figure S1. Bar blot representation of the heat-map data from Figure 2C. A) 1 h incubation; B) 24 h incubation. RFU = relative fluorescence units

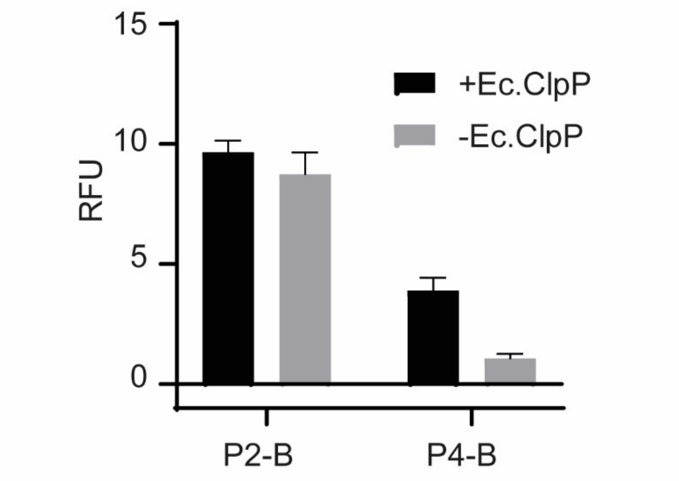

Supplementary Figure S2. Ruling out false positive hits by performing the Ec.ClpP-dependent digestion of FITC-casein in the presence or absence of Ec.ClpP. RFU = relative fluorescence units

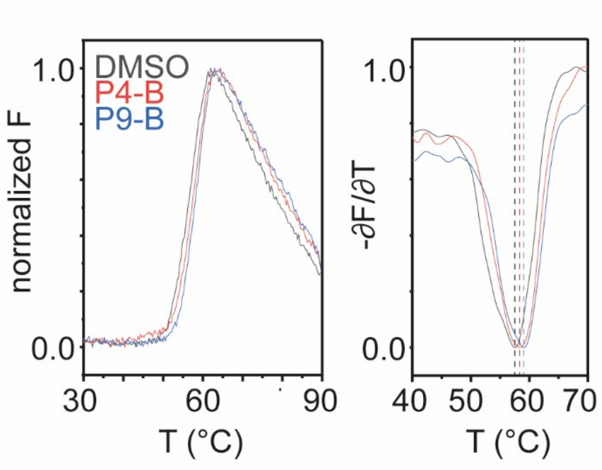

Supplementary Figure S3. Exemplary thermal shift profiles and their 1st derivative of Sa.ClpP in the presence or absence of **P4-B** and **P9-B**.

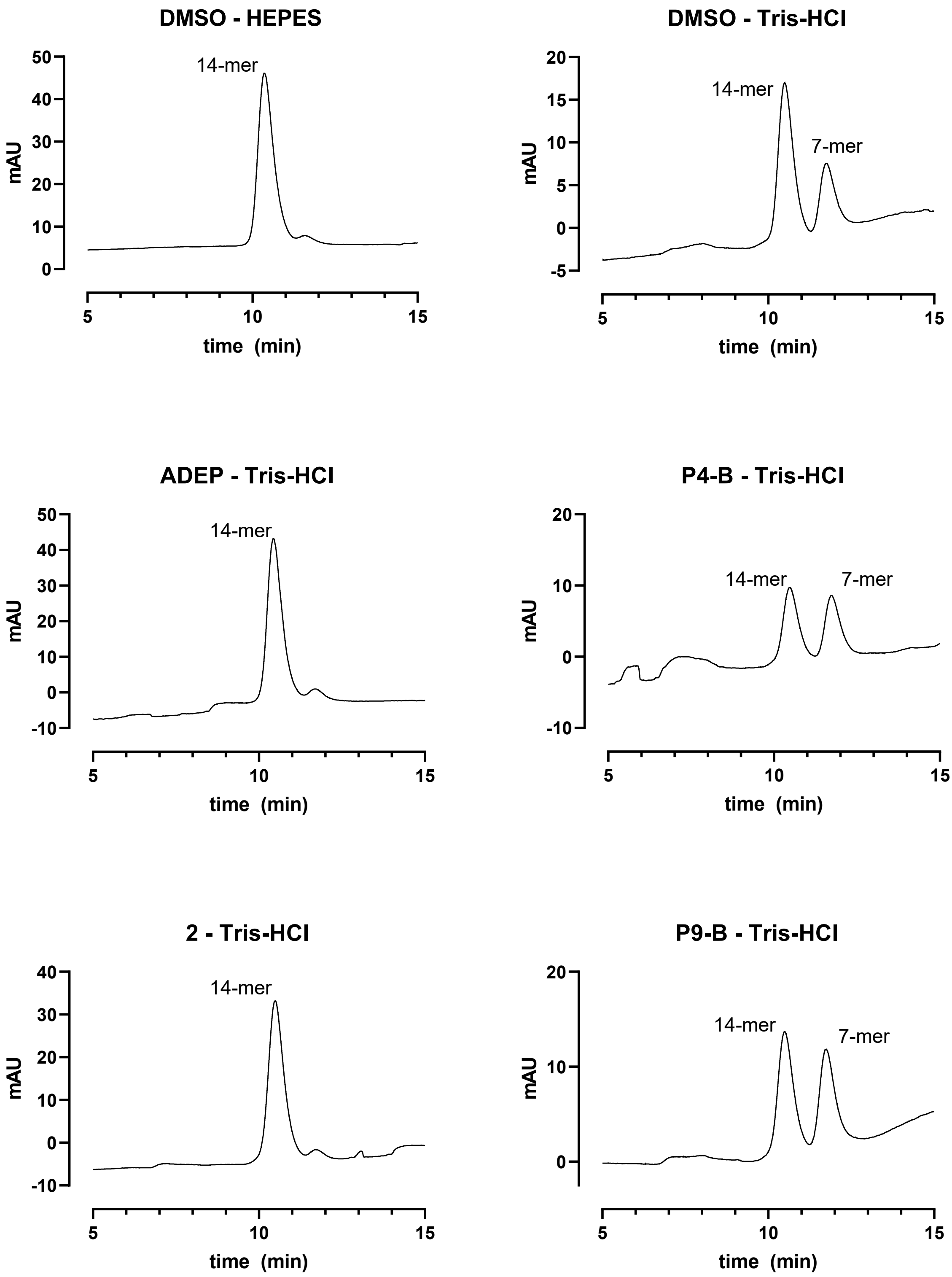

Supplementary Figure S4. Gel filtration experiments performed in stabilizing (HEPES, pH 7) and destabilizing buffer (Tris-HCl, pH 7.6) in the presence or absence (DMSO) of allosteric ligands. ADEP2 and fragment **2** are able to rescue and stabilize the tetradecameric structure of Sa.ClpP whereas **P4-B** and **P9-B** are not.

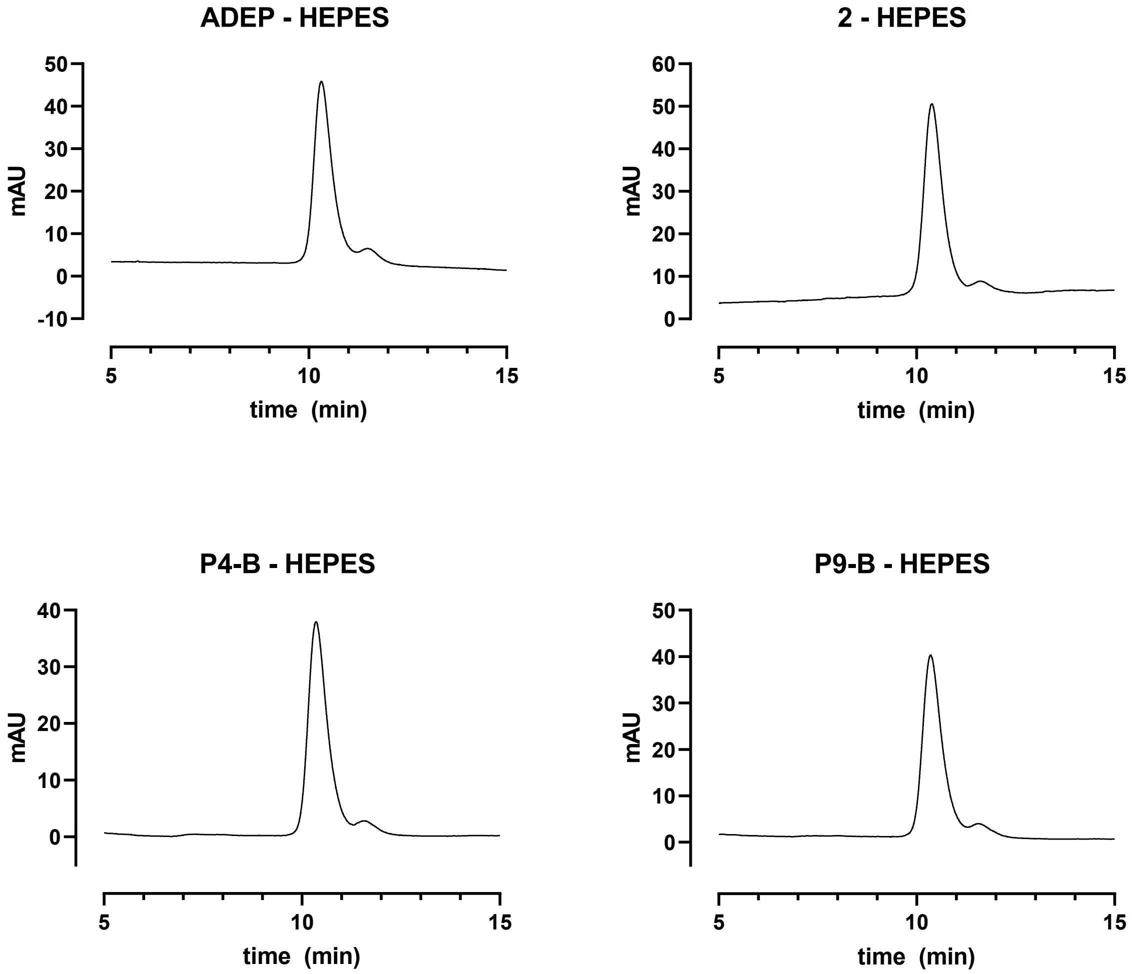

Supplementary Figure S4 continued. Gel filtration experiments performed in stabilizing (HEPES, pH 7) and destabilizing buffer (Tris-HCl, pH 7.6) in the presence or absence (DMSO) of allosteric ligands. ADEP2 and fragment **2** are able to rescue and stabilize the tetradecameric structure of Sa.ClpP whereas **P4-B** and **P9-B** are not. No active disruption of ClpP (from tetradecameric to heptameric species) is observed for any of the ligands.

METHODS AND SUPPLEMENTARY ANALYTICAL DATA

GENERAL REMARKS 6

PEPTIDE SYNTHESIS 6

PEPTIDE STAPLING 6

HPLC TRACES 9

PROTEIN PURIFICATION AND ISOLATION 24

FITC CASEIN DEGRADATION SCREEN 24

FITC CASEIN DEGRADATION RATE 25

BETA-CASEIN DEGRADATION BY EC.CLPP OR SA.CLPP 25

DOSE-RESPONSE OF P9 – B BETA-CASEIN DEGRADATION 25

FTSZ DEGRADATION BY SA.CLPP 25

THERMAL SHIFT ASSAY 25

SEC EXPERIMENTS 25

DATA ANALYSIS 27

General Remarks

All reagents were purchased from commercial vendors and used without further purification.

Constraint 1 was synthesized according to Walsh et al.[1]

1 and **2** were synthesized according to Carney et al.[2]

Peptide Synthesis

All peptides were synthesized by conventional solid-phase peptide synthesis methods (Fmoc-strategy) using HATU (4 equiv.) as coupling agent, DIPEA (8 equiv.), and amino acid (4 equiv.) in peptide-grade DMF. Fmoc-deprotection and amino acid coupling was monitored by Kaiser test.

Resin cleavage:

Resin was suspended in TFA/TIPS/EDT/H_2O (93.75/2.5/2.5/1.25) and shaken at r.t. for 3-6 h. Subsequently, the resin was filtered and washed with TFA. The filtrate was dried by a stream of N_2 overnight. The dry crude material was dissolved in minimal amounts TFA and the peptide was precipitated in ice-cold diethyl ether. The suspension was centrifuged and the peptide pellet was washed twice with ice-cold diethyl ether. The peptide pellets were dried under a stream of nitrogen gas, and stored at -20 °C until further purification by preparative HPLC.

Peptide stapling

All linear peptides were dissolved in DMSO to yield a concentration of 20 mg/mL. The staples, 1,3-dichloroacetone was dissolved in acetonitrile to yield a 10 mg/mL solution, and constraint 1 was dissolved in DMSO to yield a final concentration of 10 mg/mL. 2-iodo-acetamide was dissolved in acetonitrile to yield a 10 mg/mL solution. Peptide stapling was performed in phosphate buffer (100 mM, pH 7.4) at a peptide concentration of 2 mg/mL. Staples (1.1 equiv) and 2-iodoacetamide (2.2 equiv) were added from their respective stock solutions to the peptide solution and shaken at r.t. for 60-120 min. In case the linear peptide has oxidized to form the disulfide analogue, tris(2-carboxyethyl)phosphine (1.1 equiv.) was added to the reaction solution.

HPLC and LCMS was used to monitor the progress of the stapling reaction. Upon completion of the reaction as judged by HPLC, the reaction solutions were diluted with H_2O (5x reaction volume) and lyophilized. The dry crude reaction mixture was stored at -20 °C until preparative HPLC was used to purify the stapled peptides. HPLC purification was performed on an Agilent 1260 Infinity II system using Supelcosil™ ABZ+PLUS (5 µm, 25 cm x 21.2 mm) column at a flow rate of 20 mL/min. Gradient elution was performed with eluent A (H_2O + 0.1% TFA) and eluent B (acetonitrile + 0.05% TFA).

Peptides were characterized by analytical HPLC using an Agilent Eclipse XDB – C18 (5 µm, 4.6 mm x 150 mm) column at a flow rate of 1 mL/min, detection at 220 and 280 nm. Gradient elution was performed with eluent A (H_2O + 0.05% TFA) and eluent B (acetonitrile + 0.05% TFA). LC-MS was performed on a Waters ACQUITY H-class UPLC with an ESCi Multi-Mode ionization Water SQ-detector 2 sprectrometer using MassLynx 4.1 software. System setup: solvent A - 2 % formic acid and solvent B acetonitrile; detector: PDA detector 200-800 nm, interval 1.2 nm; column: ACQUITY UPLC CSH C18 (2.1 mm x 50 mm, 1.7 µm, 130 Å).

[1] S. J. Walsh, et al., *Chemical Science* **2019**, *10*, 694-700.
[2] D. W. Carney, et al., *ChemBioChem* **2014**, *15*, 2216-2220.

Supplementary Table S1. Summary and characterization of linear peptide precursors.

Entry		calcd. Mw [M+H]+	found	purity (%)	red:ox[a]
Peptide 1	P1	640.3	640.3	98,1	>50:01
Peptide 2	P2	711.3	711.4	95,7	22:01
Peptide 3	P3	812.3	812.4	94,2	16:01
Peptide 4	P4	697.3	697.3	90,2	>25:01
Peptide 5	P5	768.3	768.3	99,9	09:01
Peptide 6	P6	869.4	869.3	99,8	07:01
Peptide 7	P7	697.3	697.6	93,4	10:20
Peptide 8	P8	621.2	621.5	99,0	n.d.
Peptide 9	P9	733.3	733.4	99,0	n.d.

[a] as determined by peak integration of the respective peaks by analytical HPLC; n.d. = not determined. red:ox ratio of reduced and oxidized species.

Supplementary Table S2. Summary and characterization of macrocycle library.

entry	constraint	calcd. Mw $[M+H]^+$	found	isolated yield (%)	purity (%)	purity product/ox (%)	product /ox ratio
P1	A	859.4	859.4	75,6	86,6	98,2	7
	L	754.3	754.3	65,5	92,3		
P2	B	765.3	765.5	13,5	>99		
	A	930.4	930.3	25,7	83,1	95,7	7
	L	825.3	825.5	40,7	96,9		
P3	B	866.4	866.3	55,3	98,8		
	A	1031.4	1032.2	20,5	88,1	93,0	18
	L	948.6	948.6	41,8	94,0		
P4	B	751.3	751.2	27,3	97,0		
	A	916.4	917.2	6,8	89,6	89,7	1960
	L	811.3	811.4	20,5	95,3		
P5	B	822.3	822.2	48,3	>99		
	A	987.4	988.2	46,2	86,6	97,8	8
	L	882.4	882.3	52,5	>99		
P6	B	923.4	923.3	19,0	98,7	98,7	76
	A	1088.5	1088.4	34,3	87,5	97,0	9
	L	983.4	983.3	44,7	>99		
P7	B	751.3	751.4	46,6	97,3		
	A	916.4	916.7		61,0	>99	2
P8	B	675.3	675.4	18,4	>99		
	A	840.4	840.5		90,6	>99	10
P9	B	787.3	787.6	43,3	>99		
	A	952.4	952.8	14,1	98,7		78
	L	847.3	847.7	58,9	>99		

Chemical Structure of all tested compounds

P1

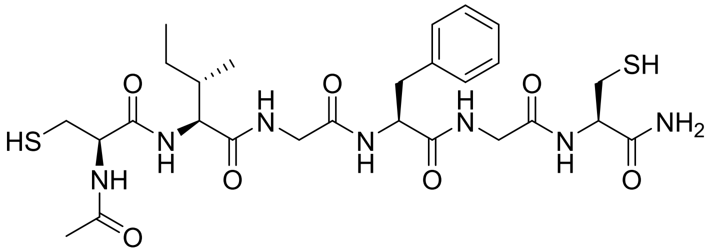

P2

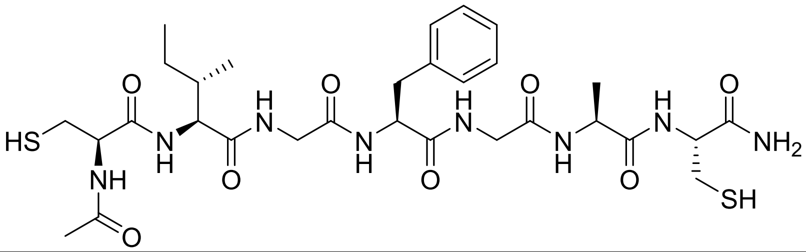

P3

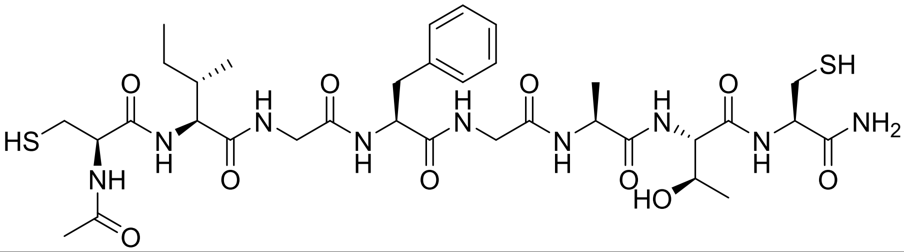

P4

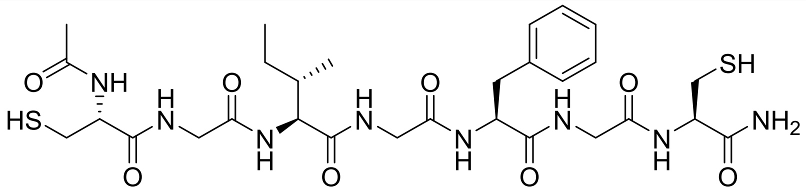

P5

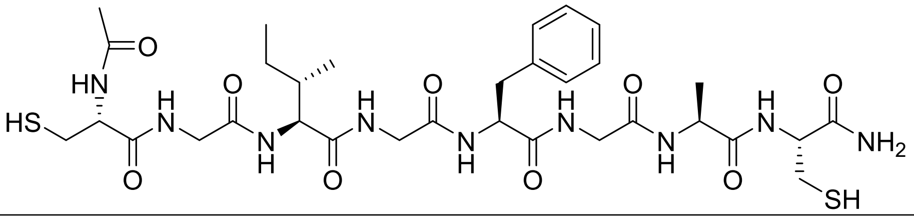

P6

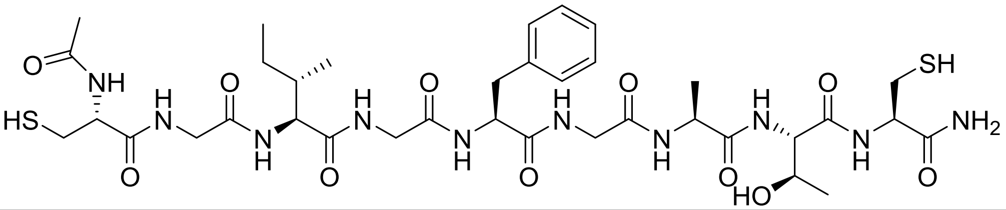

P7

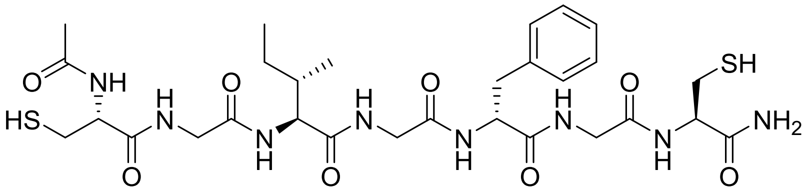

P8

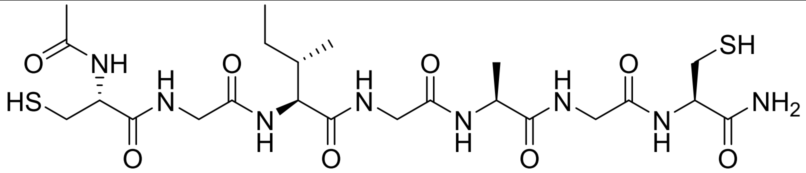

**P9**	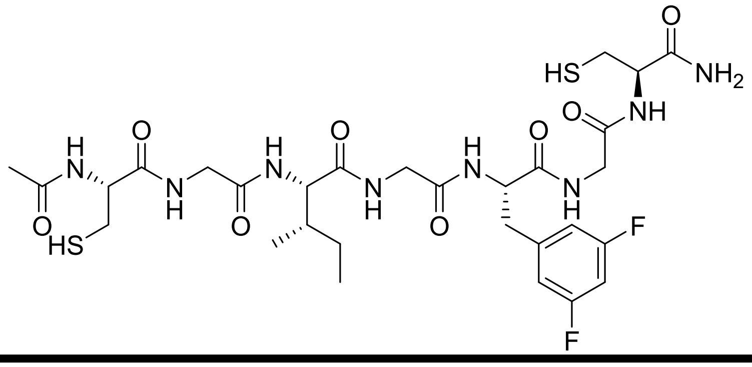

	**A**	**B**	**L**
	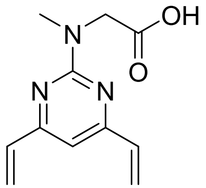	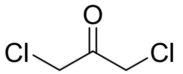	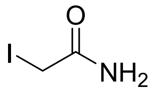
**P1**	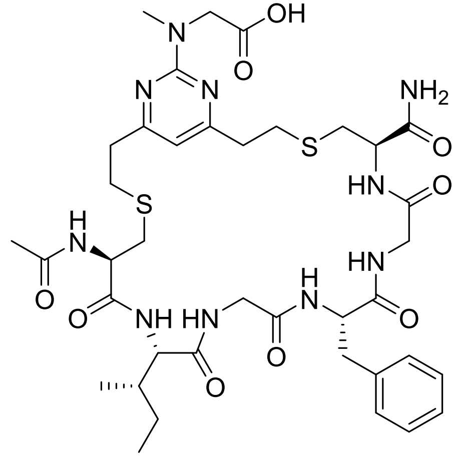	-	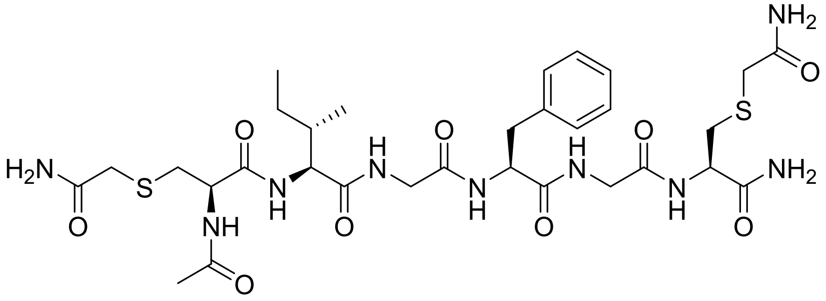

	A	B	L
**P2**	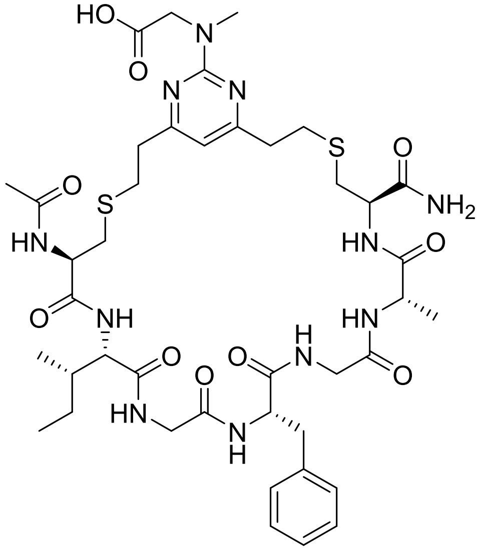	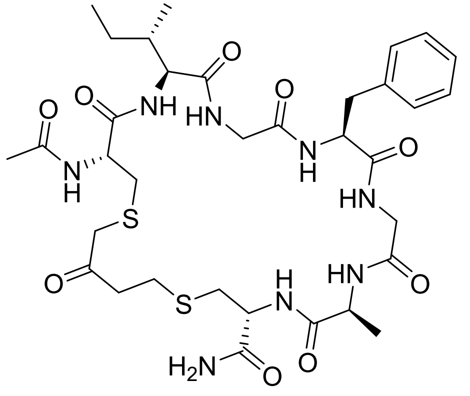	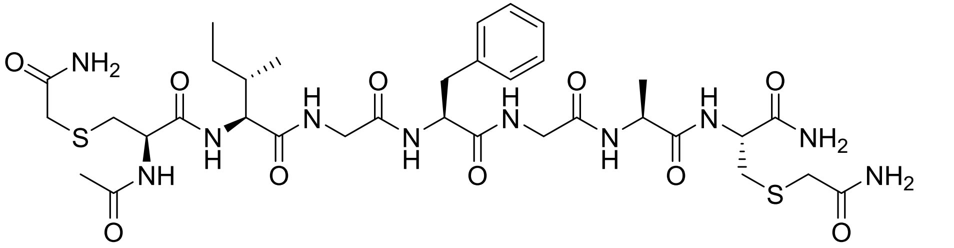
**P3**	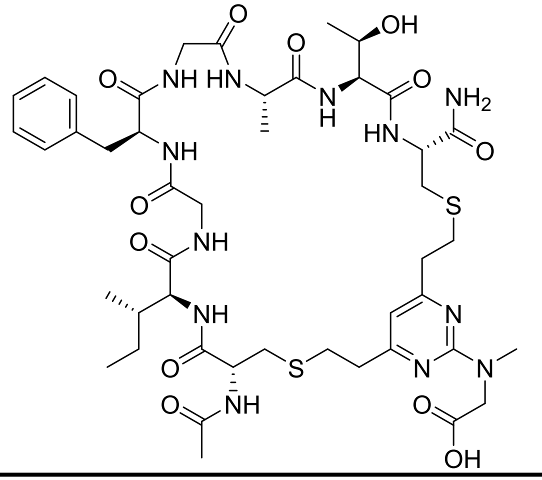	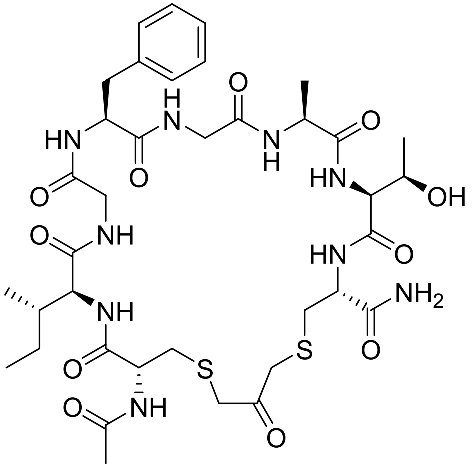	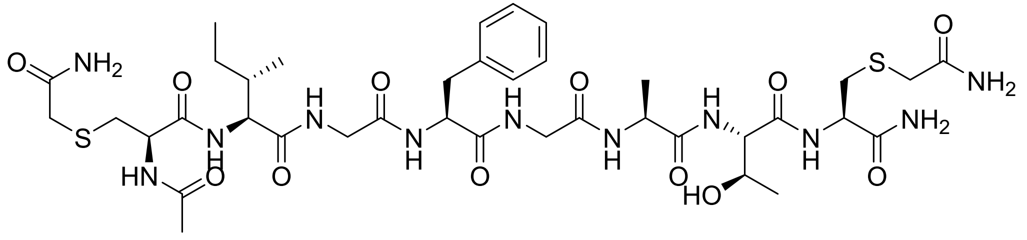

	A	B	L
**P4**	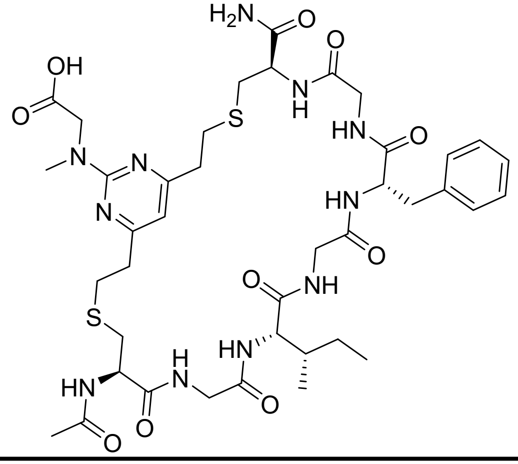	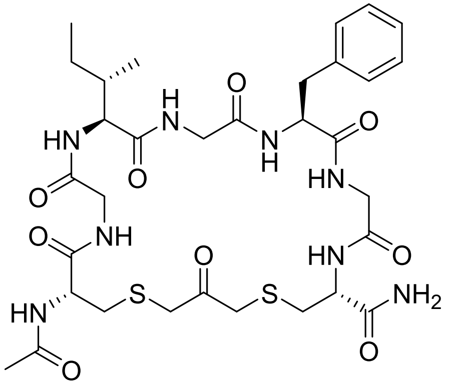	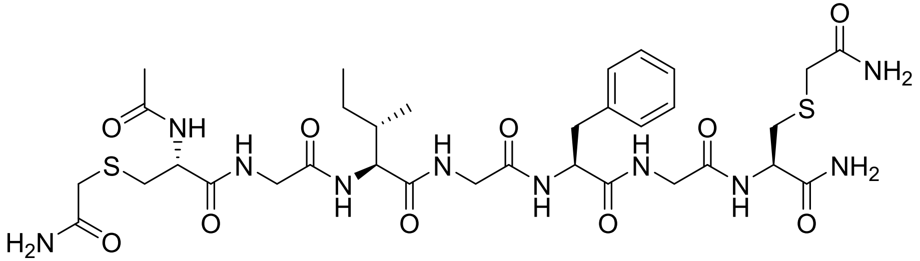
**P5**	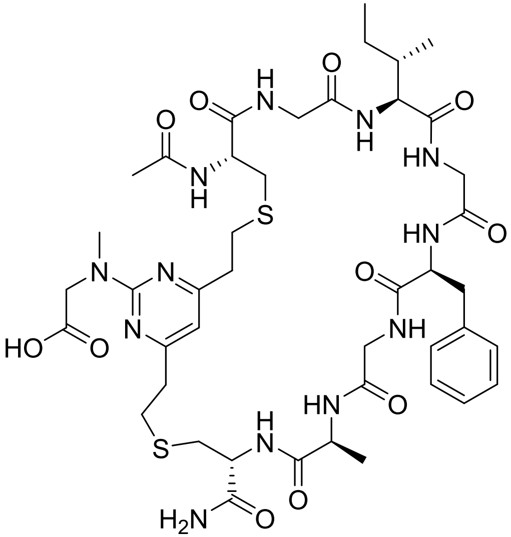	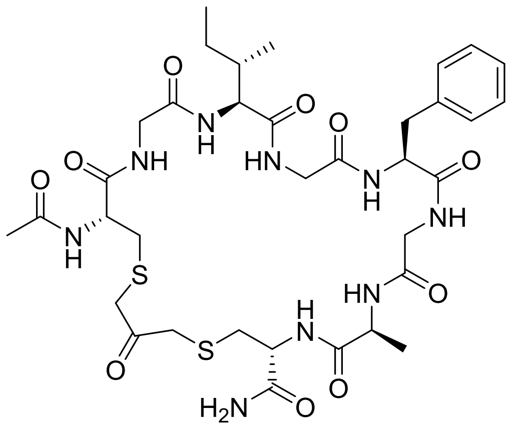	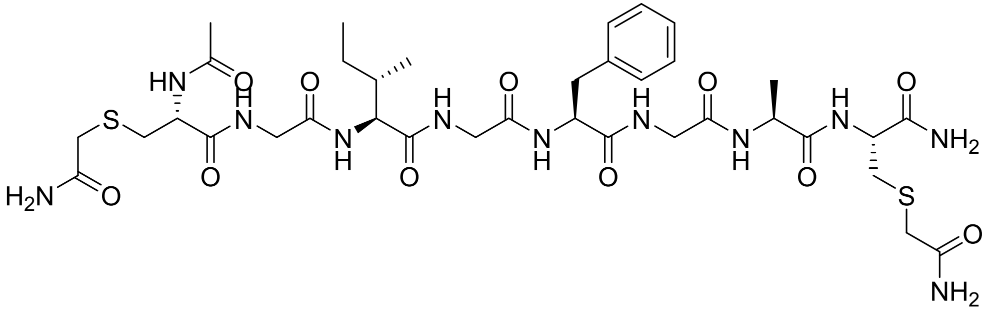

	A	B	L
**P6**	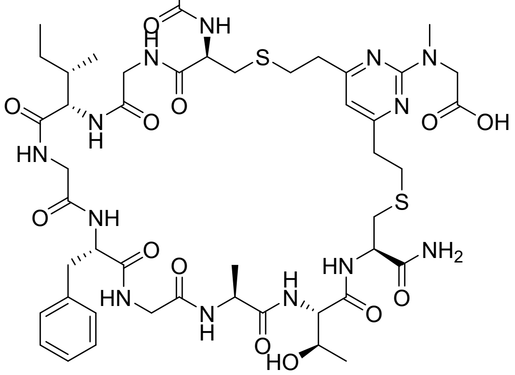	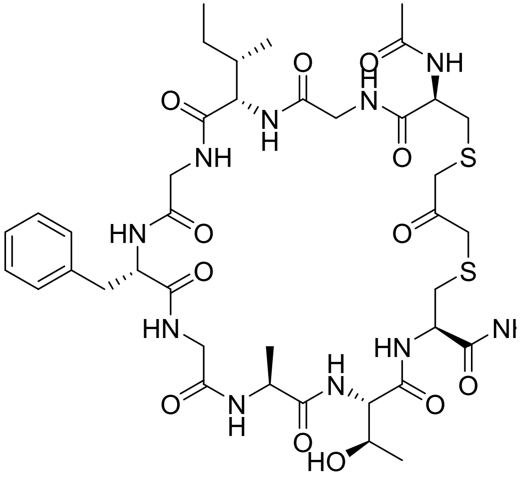	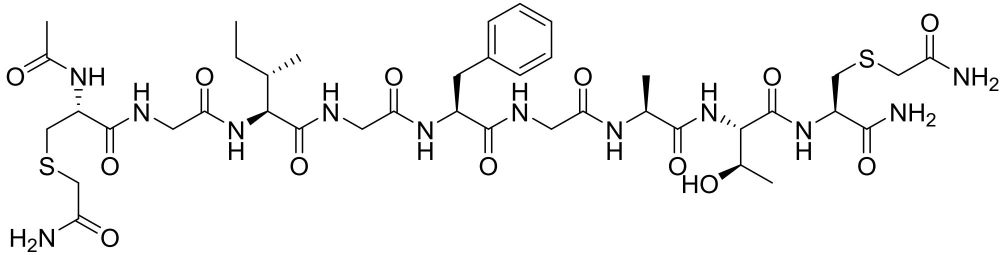
**P7**	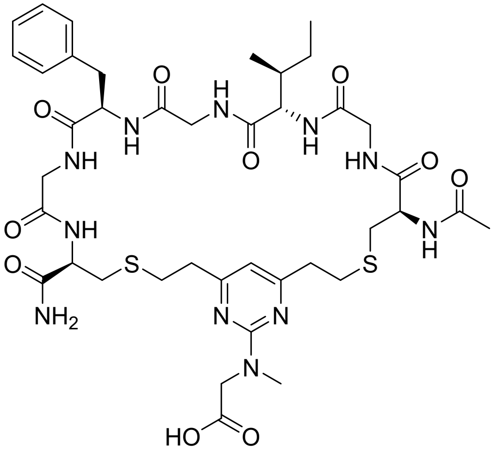	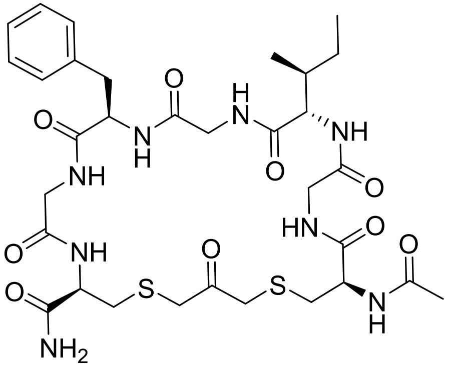	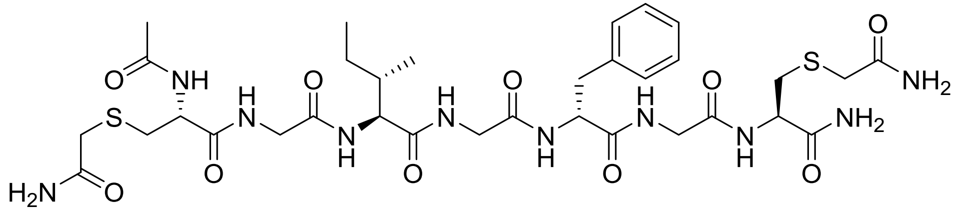

	A	B	L
**P8**	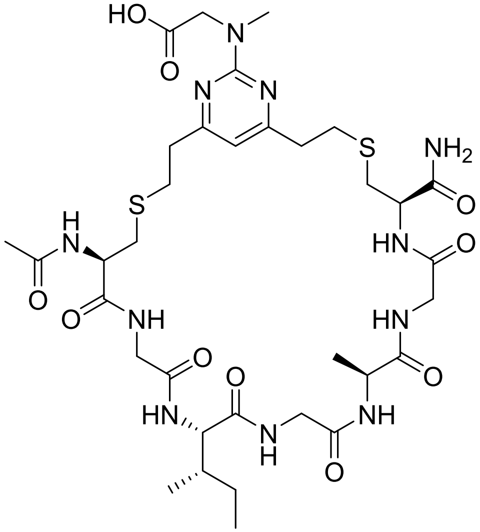	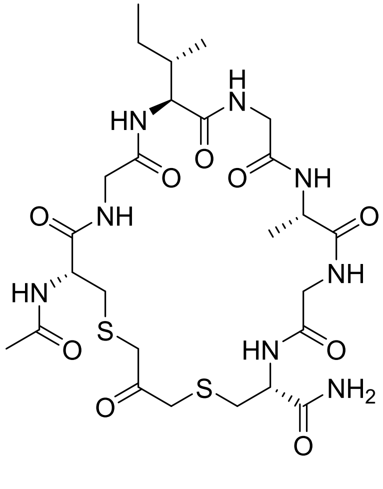	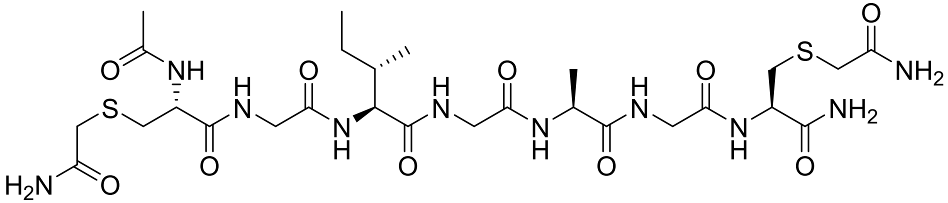
**P9**	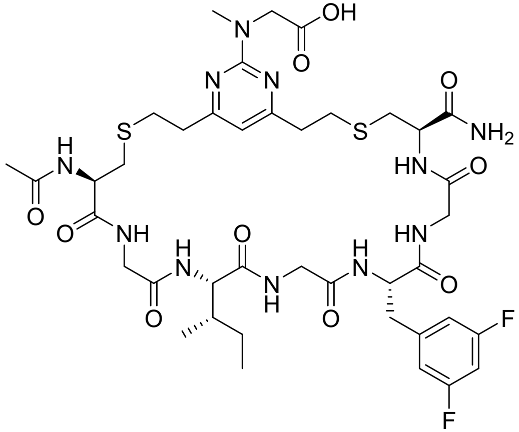	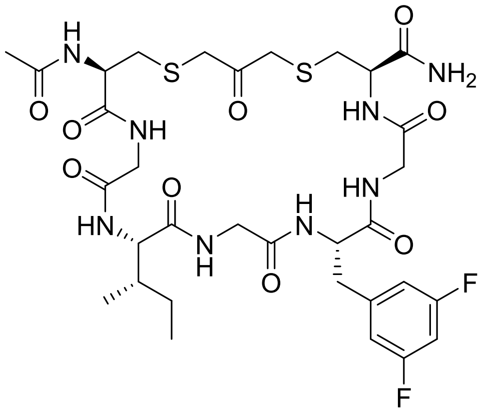	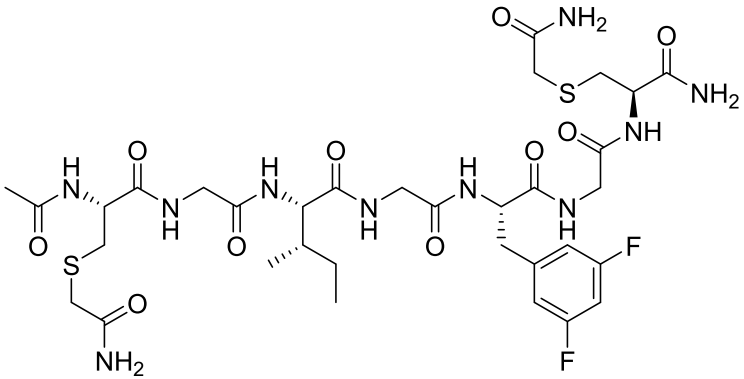

	A	B	L
**P9 S-S**	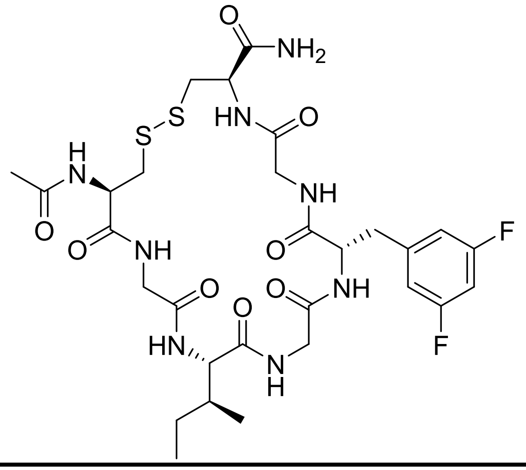O<br>NH2<br>S<br>S<br>HN<br>O<br>H<br>N<br>NH<br>O<br>O<br>NH<br>O<br>F<br>O<br>NH<br>O<br>O<br>HN<br>N<br>H<br>F	-	-

HPLC traces

Linear peptides:[3]

[3] * indicates oxidized macrocycle.

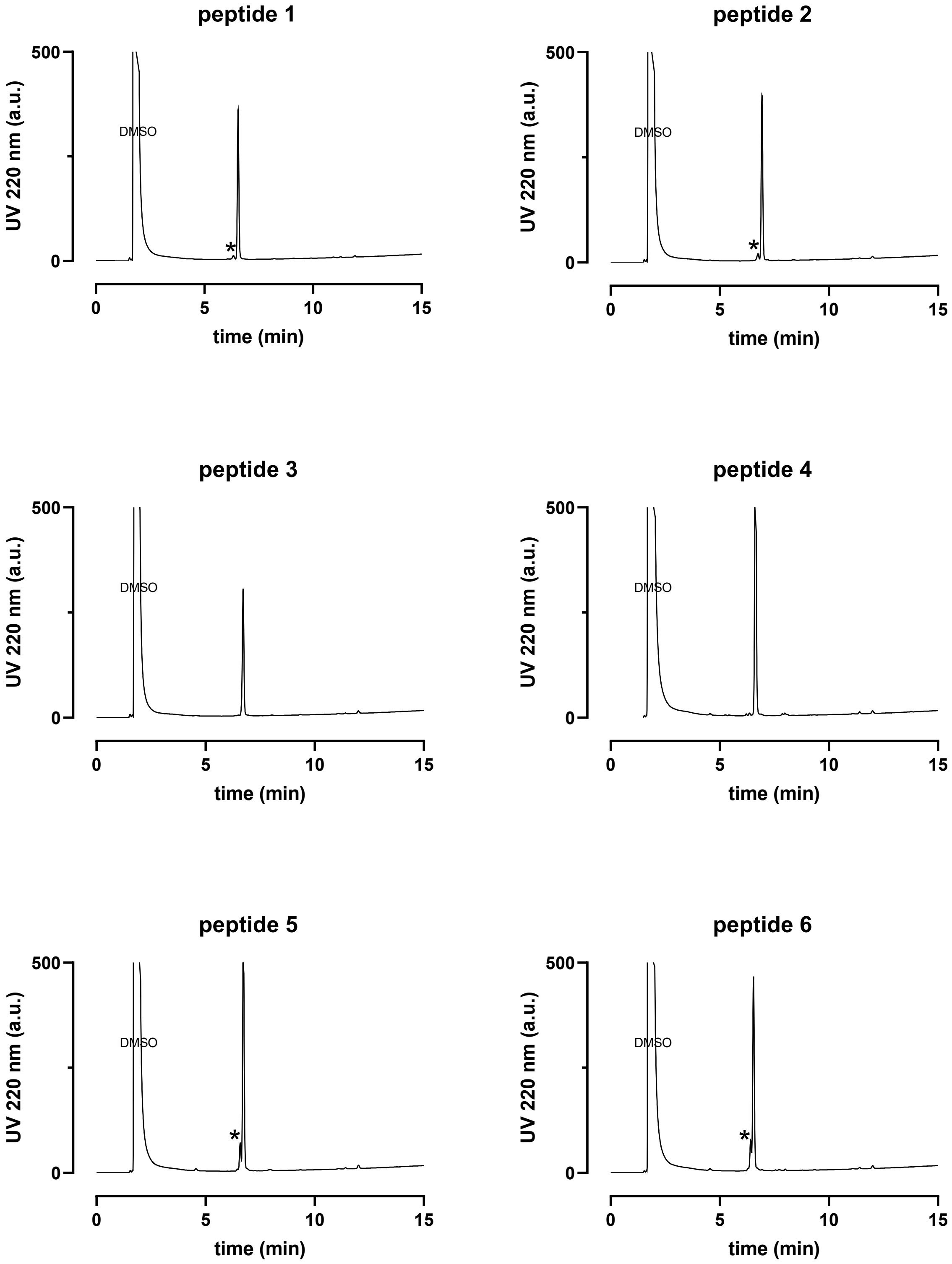
peptide 1
peptide 2
peptide 3
peptide 4
peptide 5
peptide 6
UV 220 nm (a.u.)
500
0
DMSO
*
0
5
10
15
time (min)

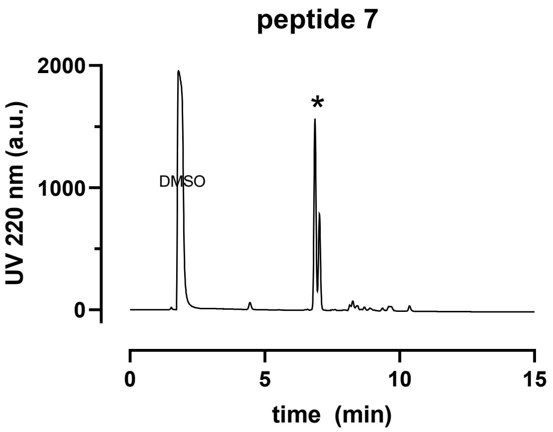
peptide 7
2000
1000
0
UV 220 nm (a.u.)
DMSO
*
0
5
10
15
time (min)

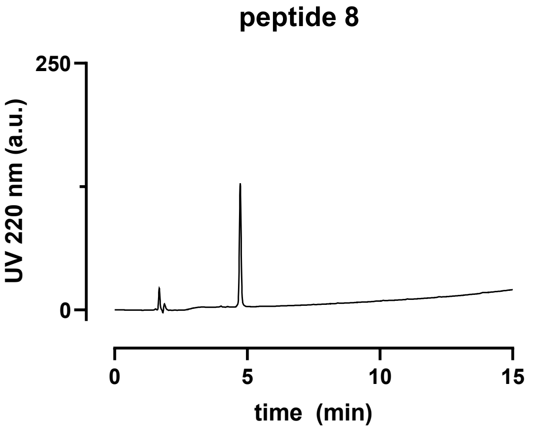
peptide 8
250
0
UV 220 nm (a.u.)
0
5
10
15
time (min)

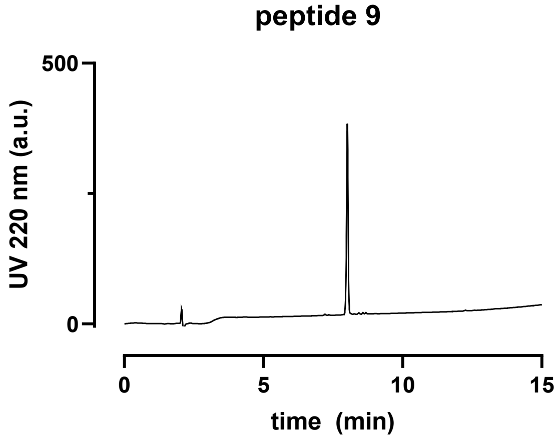
peptide 9
500
0
UV 220 nm (a.u.)
0
5
10
15
time (min)

Constrained peptides

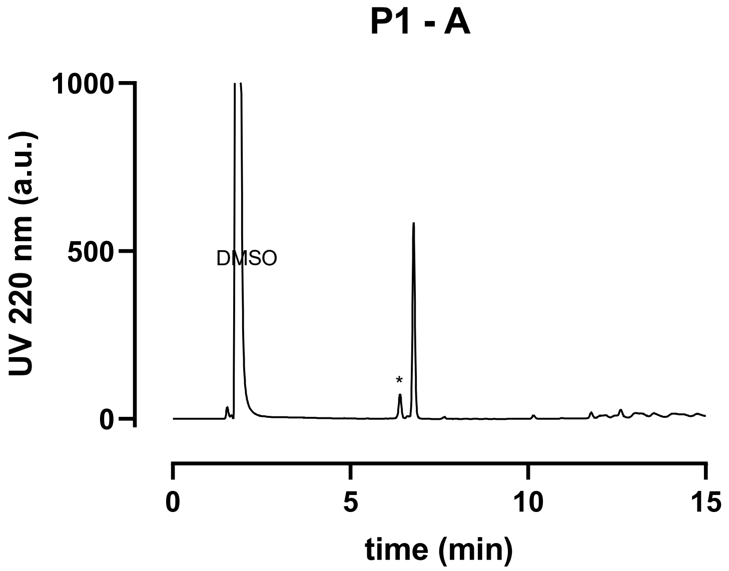

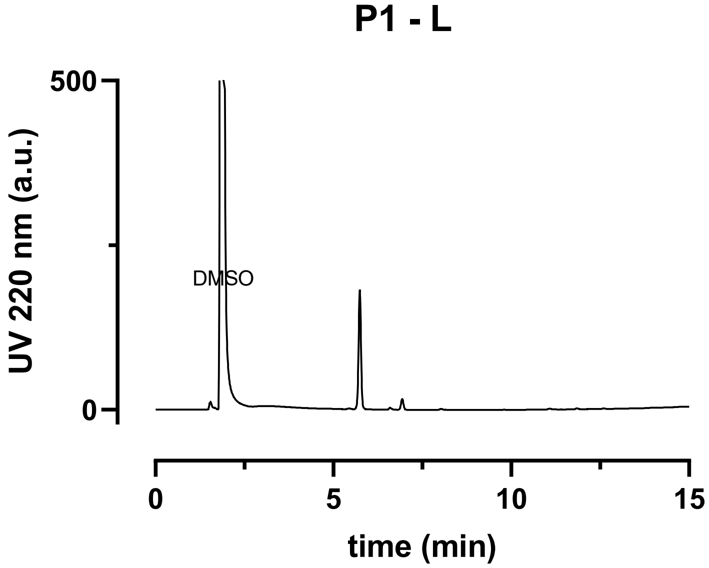

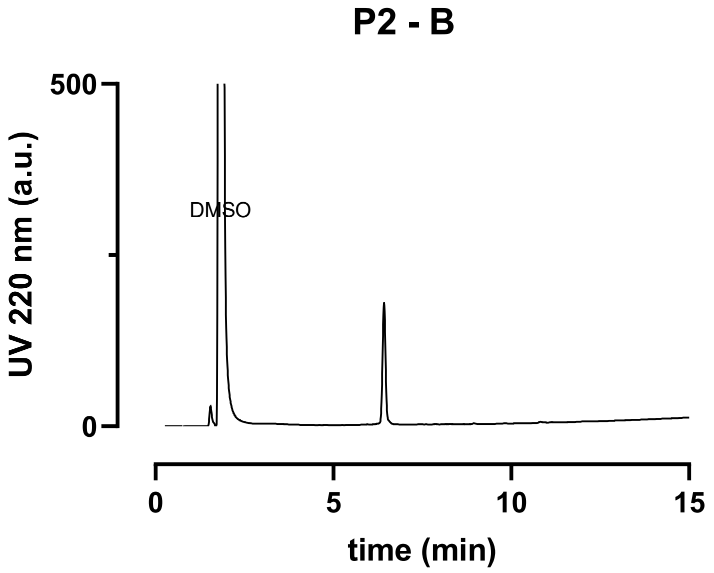
P2 - B
500
0
UV 220 nm (a.u.)
DMSO
0
5
10
15
time (min)

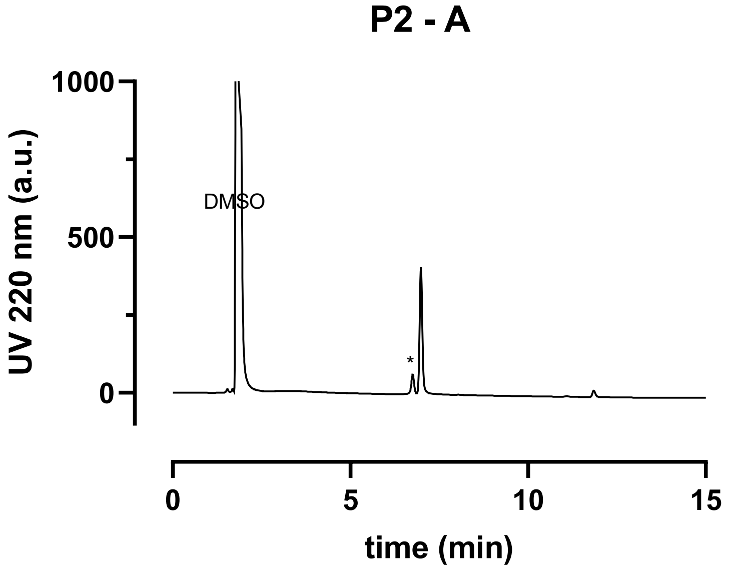
P2 - A
1000
500
0
UV 220 nm (a.u.)
DMSO
*
0
5
10
15
time (min)

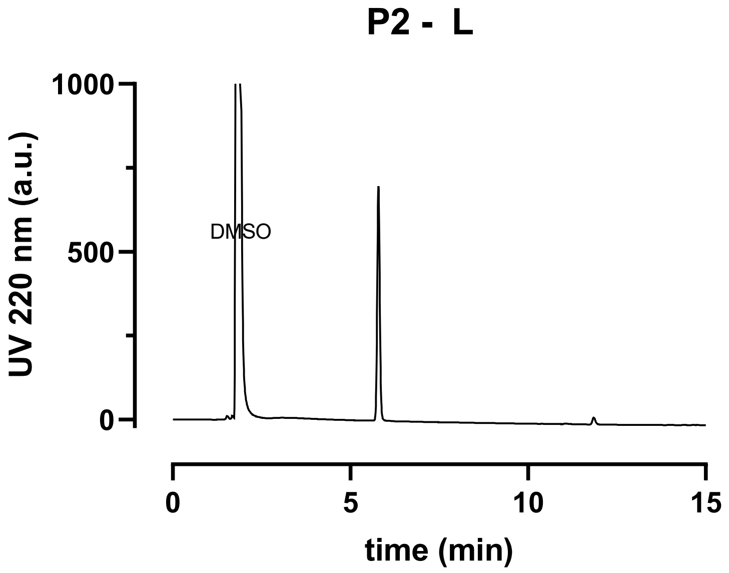
P2 - L
UV 220 nm (a.u.)
1000
500
0
DMSO
0
5
10
15
time (min)

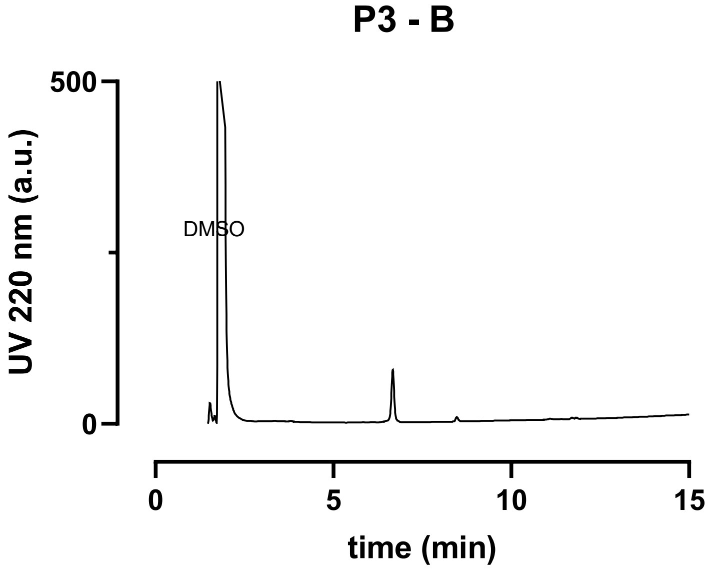
P3 - B
UV 220 nm (a.u.)
500
0
DMSO
0
5
10
15
time (min)

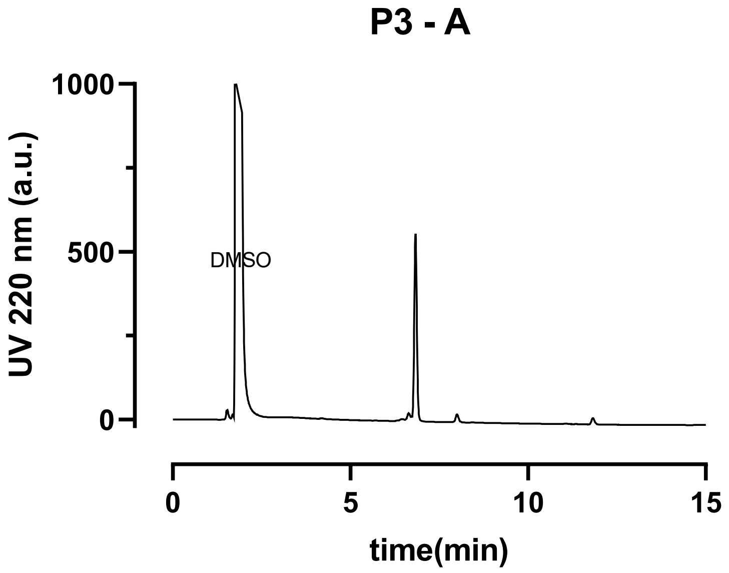
P3 - A
UV 220 nm (a.u.)
1000
500
0
DMSO
0
5
10
15
time(min)

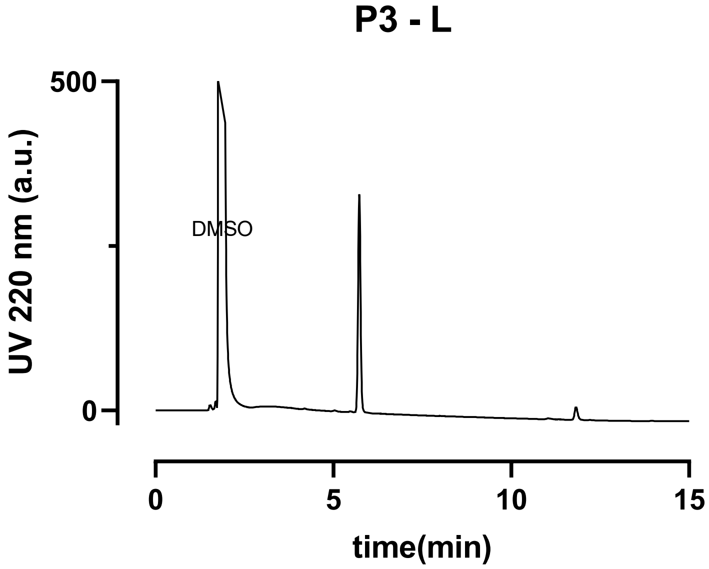
P3 - L
UV 220 nm (a.u.)
500
0
DMSO
0
5
10
15
time(min)

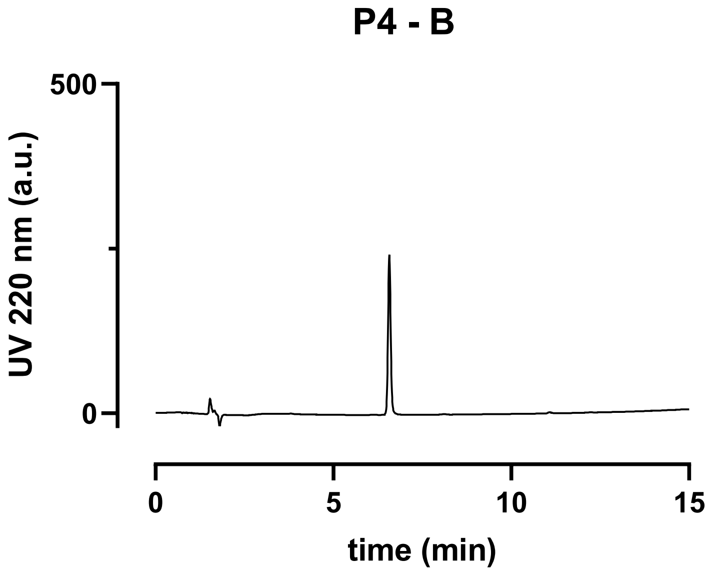
P4 - B
UV 220 nm (a.u.)
500
0
0
5
10
15
time (min)

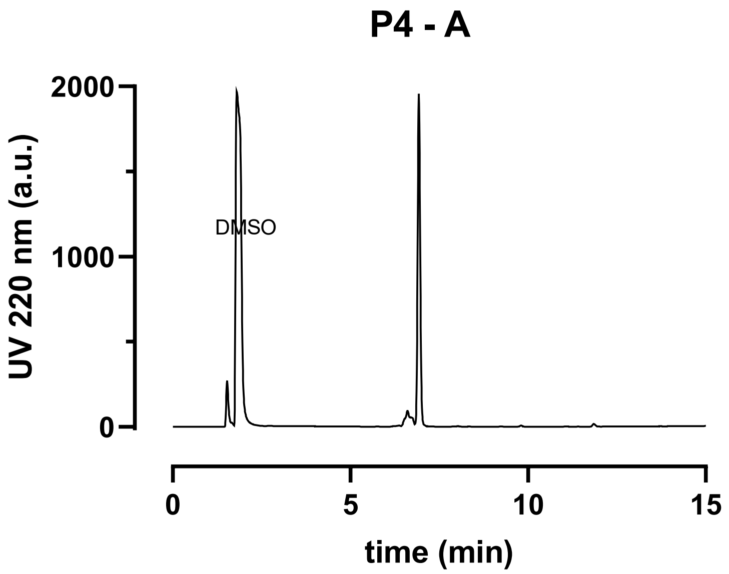
P4 - A
UV 220 nm (a.u.)
2000
1000
0
DMSO
0
5
10
15
time (min)

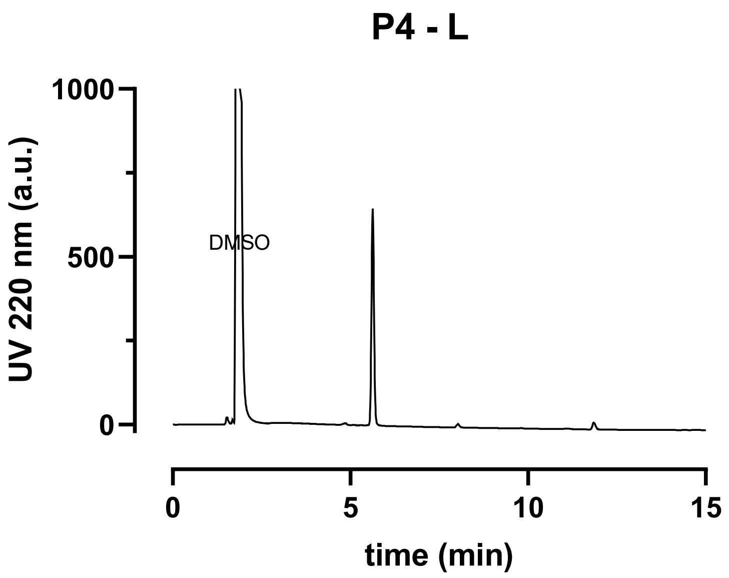
P4 - L
UV 220 nm (a.u.)
1000
500
0
DMSO
0
5
10
15
time (min)

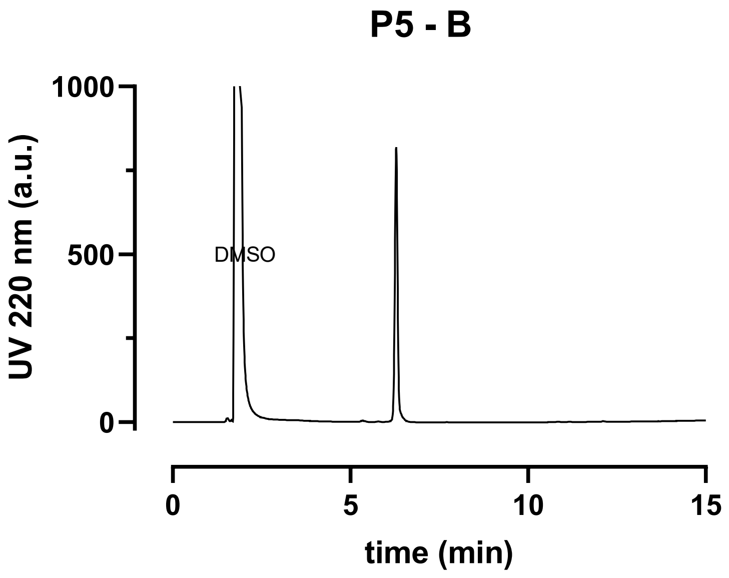
P5 - B
UV 220 nm (a.u.)
1000
500
0
DMSO
0
5
10
15
time (min)

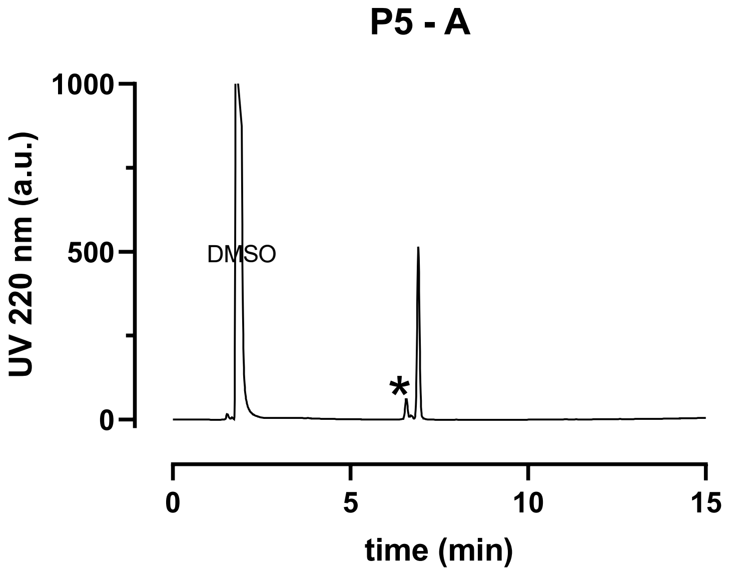
P5 - A
1000
500
0
UV 220 nm (a.u.)
DMSO
*
0
5
10
15
time (min)

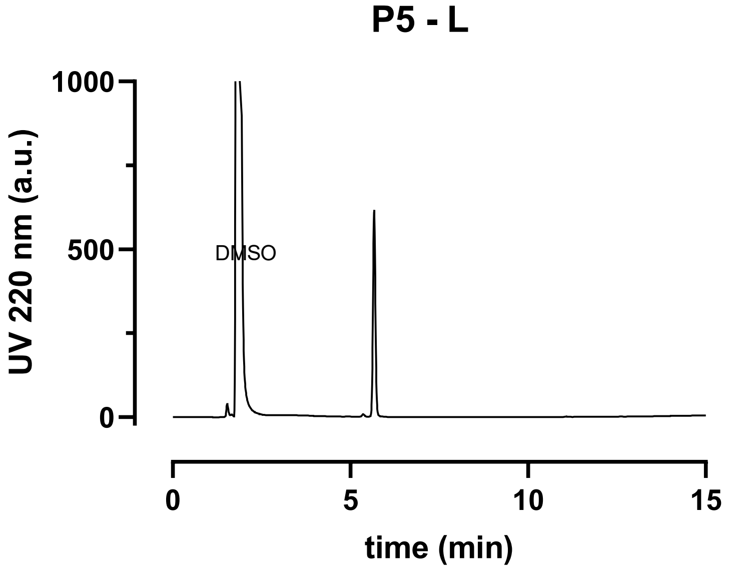
P5 - L
1000
500
0
UV 220 nm (a.u.)
DMSO
0
5
10
15
time (min)

P6 - B

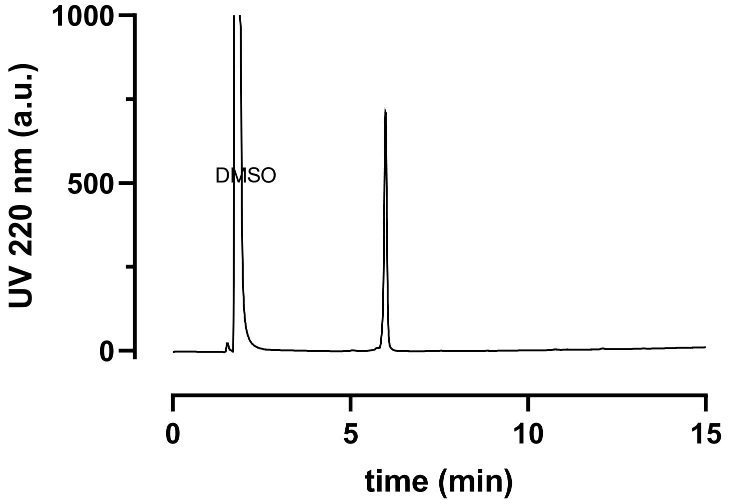
1000
500
0
UV 220 nm (a.u.)
DMSO
0
5
10
15
time (min)

P6 - A

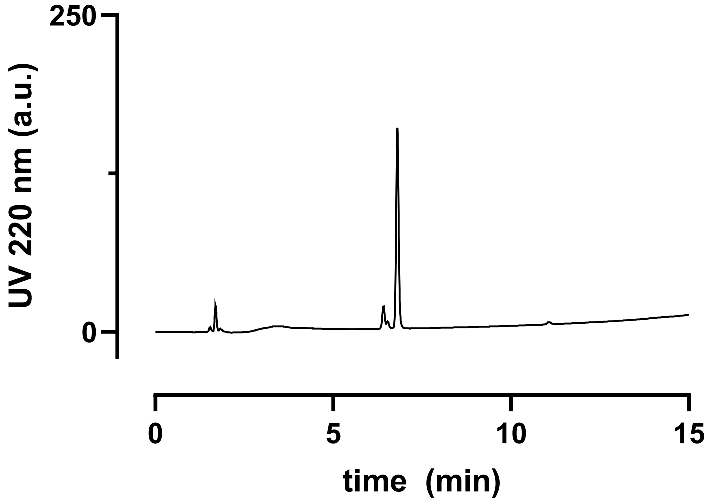
250
0
UV 220 nm (a.u.)
0
5
10
15
time (min)

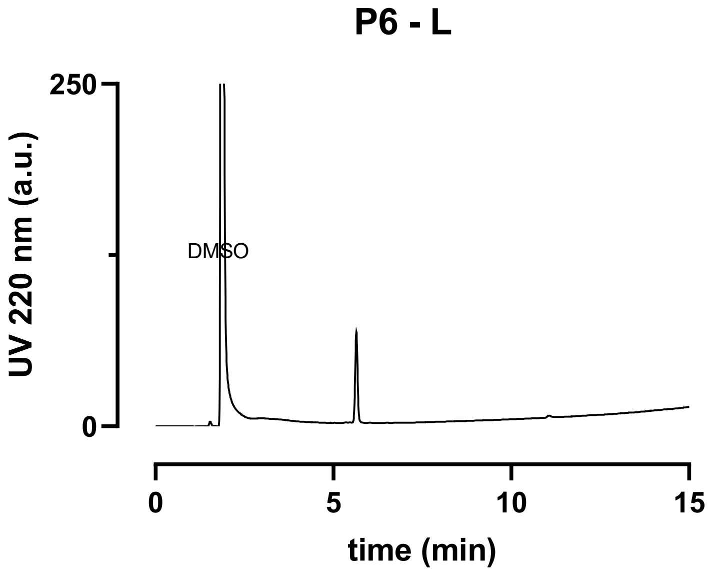
P6 - L
250
0
UV 220 nm (a.u.)
DMSO
0
5
10
15
time (min)

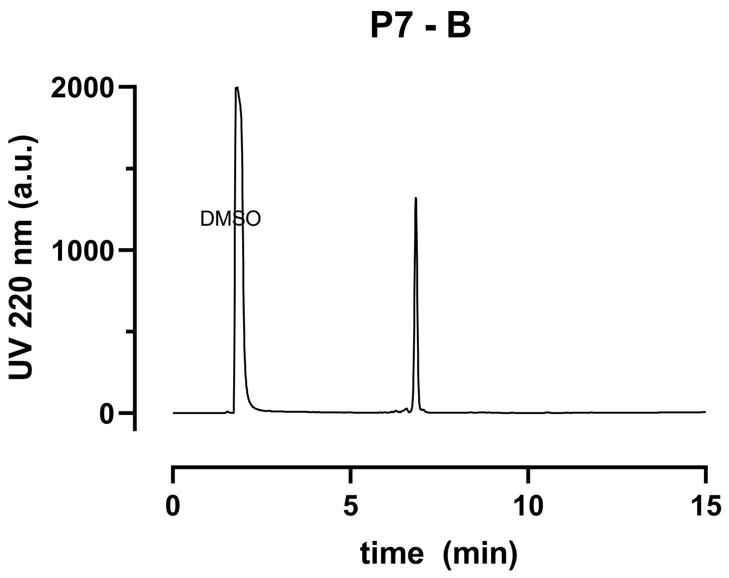
P7 - B
2000
1000
0
UV 220 nm (a.u.)
DMSO
0
5
10
15
time (min)

P7 - A

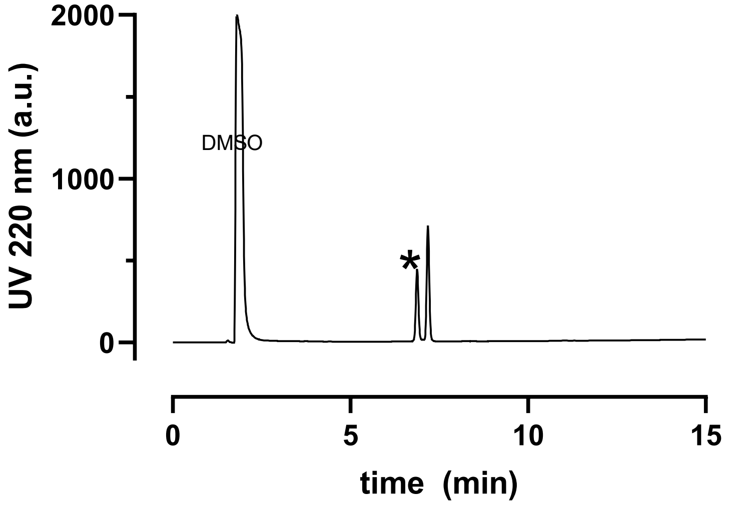
2000
1000
0
UV 220 nm (a.u.)
DMSO
*
0
5
10
15
time (min)

P8 - B

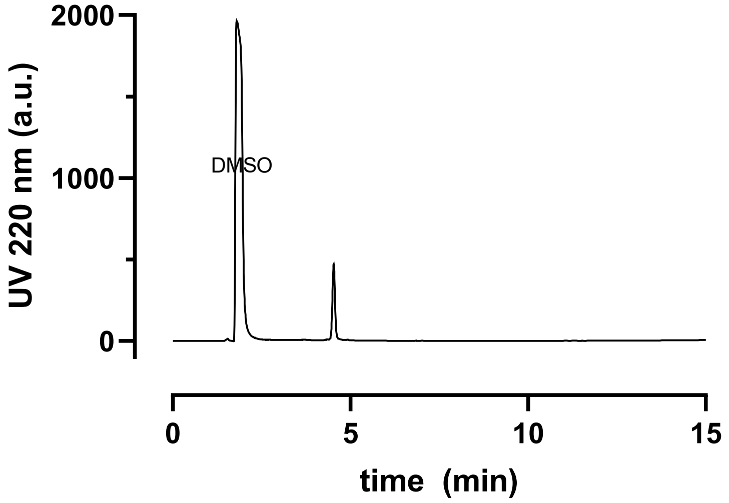
2000
1000
0
UV 220 nm (a.u.)
DMSO
0
5
10
15
time (min)

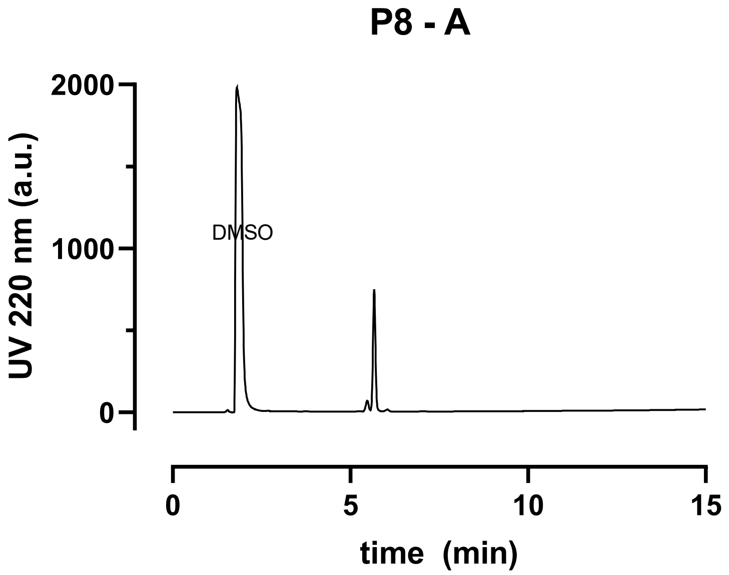
P8 - A
2000
1000
0
UV 220 nm (a.u.)
DMSO
0
5
10
15
time (min)

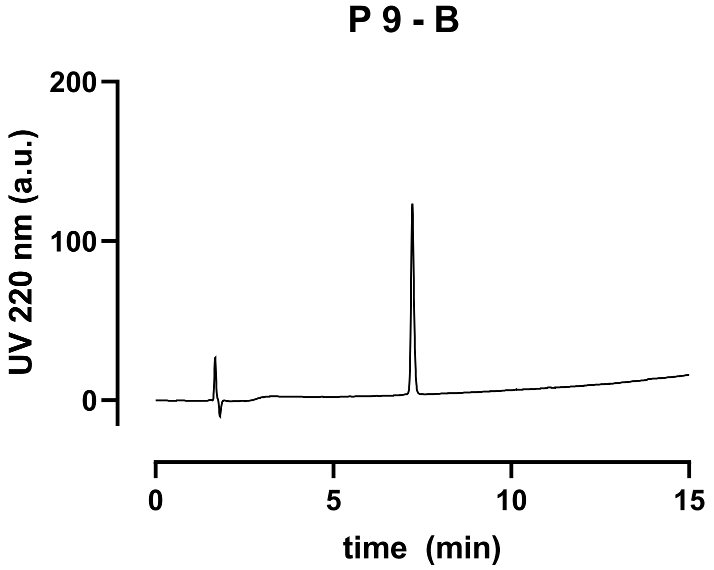
P 9 - B
200
100
0
UV 220 nm (a.u.)
0
5
10
15
time (min)

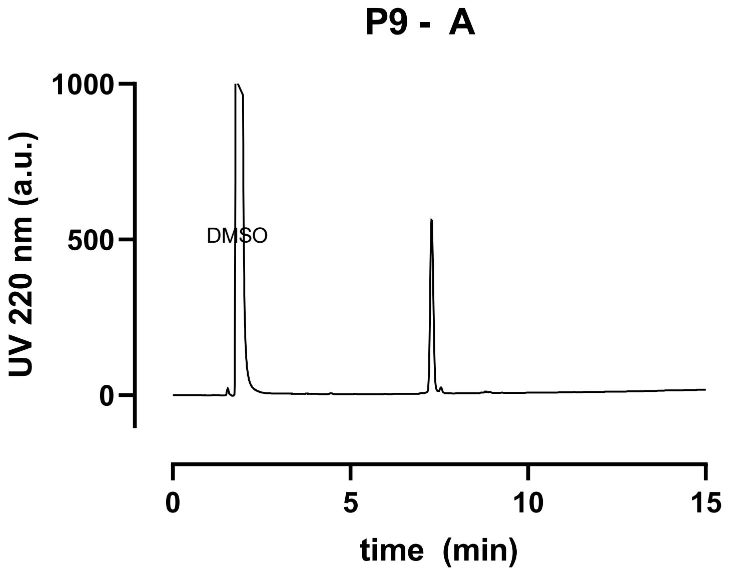
P9 - A
UV 220 nm (a.u.)
1000
500
0
DMSO
0
5
10
15
time (min)

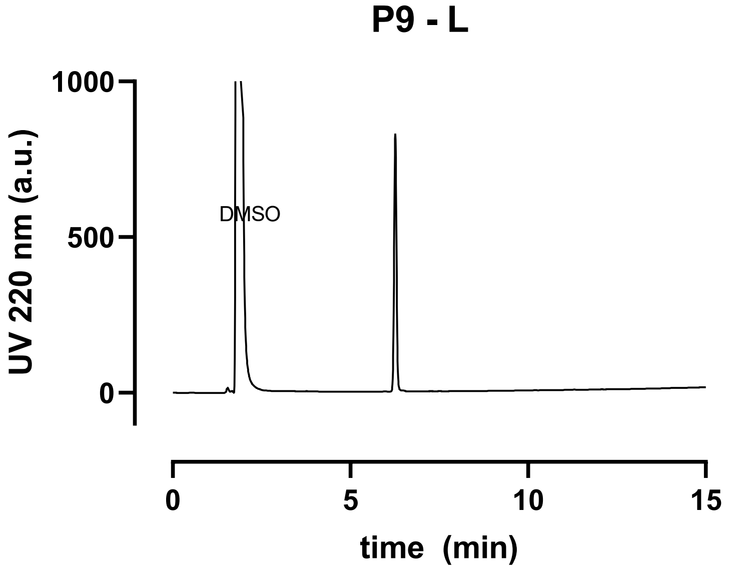
P9 - L
UV 220 nm (a.u.)
1000
500
0
DMSO
0
5
10
15
time (min)

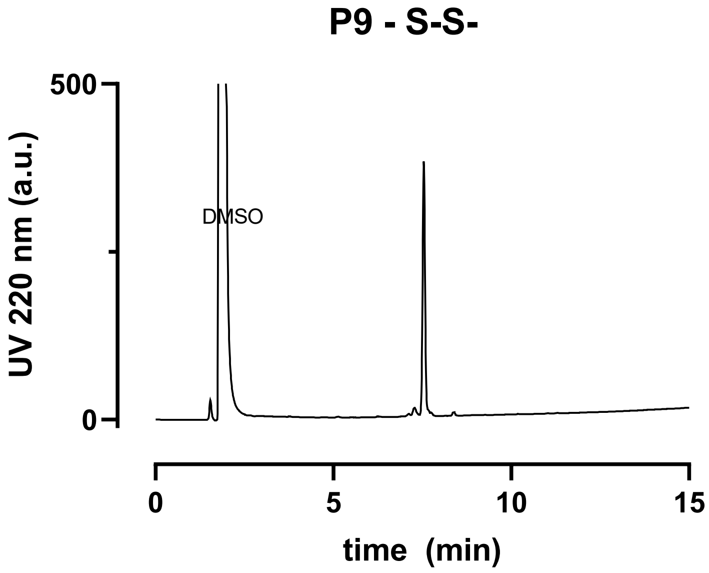
P9 - S-S-
500
0
UV 220 nm (a.u.)
DMSO
0
5
10
15
time (min)

Protein purification and isolation

Proteins were isolated essentially as described previously.[4] In short, His-tagged ClpP was expressed in *E. coli* C41 (DE3) cells. A single colony was incubated overnight in 2xYT media containing ampicillin (0.1 mg/mL) at 37 °C. Subsequently 10 mL of the overnight inoculum was added to 1 L of 2xYT media containing ampicillin (0.1 mg/mL) and cultures were grown to OD_{600} of 0.4-0.6 and expression was induced with IPTG (0.1 mM) for 5 hours at 26 °C. Bacteria were harvested by centrifugation, the pellet was resuspended in lysis buffer (phosphate buffer pH 8 50 mM, 1 M NaCl, 5 mM imidazole, glycerol 10 % (v/v)) and bacterial cells were lysed by sonication. The suspension was cleared by centrifugation and the supernatant was directly applied to a Ni-NTA affinity column (GE Healthcare). The column was washed with an increasing gradient of buffer (sodium phosphate pH 8 50 mM, 1 M NaCl, glycerol 10 % (v/v)) containing 20-200 mM imidazole, and protein was eluted with 500 mM imidazole. Protein containing fraction were pooled and the buffer exchanged to another buffer (Tris-HCl pH 8 50 mM, 50 mM KCl, 10 mM $MgCl_2$, glycerol 10 % (v/v)). The pure protein sample was obtained after size exclusion chromatography on a HiLoad 26/600 Superdex 75 column equilibrated with buffer (Tris HCl pH 8 50 mM, 50 mM KCl, 10 mM $MgCl_2$, glycerol 10 % (v/v)), protein containing fractions were pooled, concentrated and the buffer exchanged to storage buffer (50 mM Tris-HCl pH 7.6, 1 mM DTT, 0.5 mM EDTA, 100 mM KCl, 10 % glycerol).

C-terminally Strep-tagged *S. aureus* ClpP was expressed in *E. coli* SG1146a which lacks endogenous ClpP. Therefore, 10 ml of an overnight culture was transferred into 1 L of LB medium containing ampicillin (0.1 mg/mL) and the culture grown to an OD_{600} of 0.4-0.6. Subsequently, protein expression was induced by adding IPTG (1.0 mM). After 18 hours of growth at 25 °C, cells were harvested by centrifugation and the cell pellet resuspended in buffer (150 mM NaCl, 100 mM Tris-HCl, 1 mM EDTA, pH 8). Cells were lysed by sonication und the cell lysate cleared by centrifugation. Purification of *S. aureus* ClpP was conducted via affinity chromatography on an Äkta Start system using a 5 ml StrepTrap XT column (Cytiva). Here, the protein was eluted with an isocratic gradient of buffer containing 50 mM biotin. Protein was concentrated and buffer exchanged to storage/activity buffer (100 mM NaCl, 20 mM HEPES, pH 7) using Pierce protein concentrators.

The protein concentration was determined by the Pierce BCA protein assay and calibrated with bovine serum albumin as standard according to the manufacturer's instruction. Aliquoted protein stocks were stored in storage buffer at -20 °C. For long term storage, samples were stored at -80 °C. Care should be taken to reduce the number of freeze-thaw cycles to avoid aggregation.

FITC casein degradation screen

All concentrations are given as final concentrations. Ligands were incubated at 200 µM in the presence of *E. coli* ClpP (0.5 µM), FITC-casein (Sigma-Aldrich – catalogue number C3777, 1.8 µM) and beta-bovine casein (Sigma-Aldrich – catalogue number C6905, 4.8 µM) in Tris HCl buffer (100 mM, pH 7.6) with KCl (100 mM) for 24 h at 37 °C upon which fluorescence was measured (exc. 483 nm, em 530 nm) on a CLARIOstar (BMG Labtech). Data is represented as mean ± SD of duplicates of at least two independent experiments. The mean of DMSO-treated samples is subtracted from all samples.

In parallel, all samples were incubated in the absence of *E. coli* ClpP in order to rule out false-positives.

[4] E. M. Sivertsson, et al., *Scientific Reports* **2019**, *9*, 2421.; I. T. Malik, et al., *International Journal of Molecular Sciences* **2022**, *23*.

FITC casein degradation rate

All concentrations are given as final concentrations. Ligands were incubated at 200 µM in the presence of *E. coli* ClpP (1.0 µM), and FITC-casein (Sigma-Aldrich – catalogue number C3777, 10 µM) in Tris HCl buffer (100 mM, pH 7.6) with KCl (100 mM) for up to 2 h at 37 °C reading the fluorescence (exc. 483 nm, em 530 nm) in 60 s intervals. The linear part of the fluorescence curve was used to determine the relative degradation rate. Data analysis was performed by OriginPro software. Data is represented as mean ± SD of duplicates of at least three independent experiments.

Beta-casein degradation by Ec.ClpP or Sa.ClpP

All concentrations are given as final concentrations. Ligands were incubated at 200 µM in presence of *E. coli* ClpP or *S. aureus* ClpP (1.0 µM), and bovine beta-casein (Sigma-Aldrich – catalogue number C6905, 5.0 µM or 3 µM) in Tris HCl buffer (100 mM, pH 7.6) with KCl (100 mM) or HEPES buffer (100 mM, pH 7) with NaCl (150 mM) at 37 °C. Aliquots were drawn at the indicated time points, quenched with SDS-loading buffer and heated to 90 °C for 5 min. Beta-casein digestion was visualized by non-reducing Tris-glycine SDS-PAGE with 12 % acrylamide and 4 % stacking gel or reducing Bolt 4-12% Bis-Tris Plus gels. Gels were run at constant voltage (160-200 V) for 60 min and visualized with Coomassie brilliant blue dye. Protein bands were quantified by densitometry using ImageJ from at least three independent experiments.

Dose-response of P4 – B and P9 – B beta-casein degradation

A two-fold dilution series in DMSO was performed to keep DMSO content identical. Highest concentration was 200 µM. The results from densitometric analysis were plotted in Graphpad Prism and a non-linear fit was applied to estimate the EC_{50}. Data is represented as mean ± SD of three independent experiments.

FtsZ degradation by Sa.ClpP

Sa. FtsZ[5] digestion was performed identical to the beta-casein degradation assay using Sa. FtsZ at a final concentration of 4.0 µM. Evaluation and analysis of digestion was identical to the beta-casein degradation assay.

Thermal shift assay

S. aureus ClpP (5 µM) was incubated with 200 µM of the respective ligand in HEPES buffer (100 mM, pH 7) with NaCl (150 mM) and sypro orange (Thermo Fisher Scientific, 5x) and fluorescence was monitored over a range of temperature (30-90 °C in 0.5 °C increments) on a real-time qPCR (Bio-Rad CF X96). **2** (100 µM) and DMSO served as positive and negative control, respectively. Data was plotted and analyzed by Origin-Pro and represents the mean ± SD of duplicates of at least two independent experiments.

SEC experiments

Sa.ClpP (1 mg/mL) was incubated in stabilizing or destabilizing buffer overnight at 32 °C in the presence or absence of ligands (200 µM) and subsequently analyzed by gel-filtration on pre-equilibrated Superdex 200 Increase 10/300 GL column (Cytiva). Stabilization of Sa.ClpP in HEPES buffer (100 mM, pH 7) with NaCl (150 mM) and destabilization in Tris HCl (100 mM, pH 7.6) with 100 mM KCl was established previously[6].

[5] N. Silber, et al., *mBio* **2020**, *11*, e01006-01020.
[6] I. T. Malik, et al., *International Journal of Molecular Sciences* **2022**, *23*.

Molecular weights were determined by a standard curve. Superdex 200 Increase 10/300 GL column was calibrated with low molecular weight protein standards (Cytiva) and the elution volume of each protein noted.

Supplementary Table S3. Determination of standard curve for SEC experiments.

**column specs**	**Vc [mL]**	24,00
	**Vo [mL]**	8,05

	Ve [mL]	Mw [kDa]	log_{10}(Mw)	Kav
**Thyroglobulin**	8,727	669	2,83	0,04
**Ferritin**	9,940	440	2,64	0,12
**Aldolase**	11,929	158	2,20	0,24
**Conalbumin**	13,738	75	1,88	0,36
**Ovalbumin**	14,693	44	1,64	0,42

Vc, Vo, and Ve stand for column volume, void volume, and elution volume, respectively.

The molecular weight for the observed protein species was calculated according to the following formula:

$$Kav = \frac{(Ve - Vo)}{(Vc - Vo)}$$

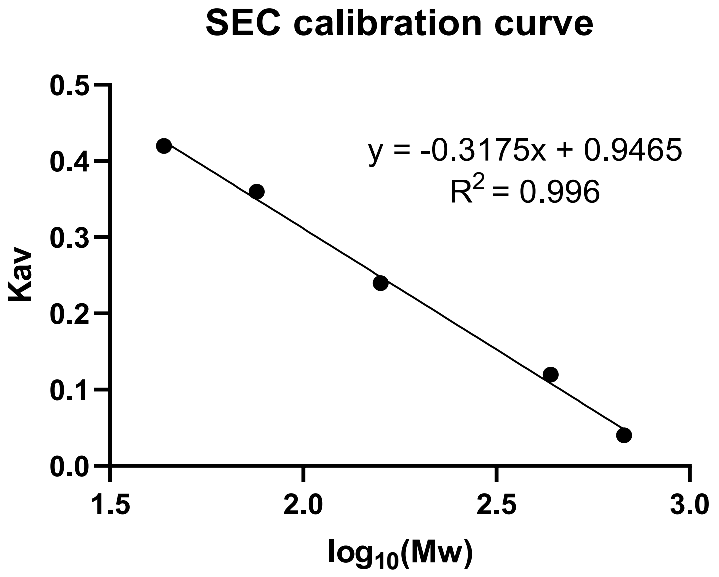

	Ve [mL]	Kav	log_{10}(Mw) observed	Mw [kDa] observed	Mw [kDa] calcd.
**14-mer ClpP**	10,490	0,15	2,50	316	316
**7-mer ClpP**	11,758	0,23	2,25	176	158

Docking procedure

Initial structures of ADEP7, ACP1, P4-B, P7-B, P8-B and P9-B were generated by Marvin 17.21.0, Chemaxon (https://www.chemaxon.com). Structures were minimized using the Steepest Decent algorithm and the UFF force field implemented in Avogadro 1.2.0[7]. The structure of ClpP used in the docking procedure was that of *E. coli* ClpP (PDB ID 1yg6).[8] A dimer of the neighbouring chains A and G in the PDB entry were extracted from one heptameric ring of ClpP and used for docking procedures.

AutoDOCK Vina in UCSF Chimera 1.15[9] was used to dock the flexible conformations of the six compounds into the structure of ClpP, which was kept rigid. Initial docking was performed with ADEP7 to identify the binding region of interest. Subsequently the receptor search volume parameters were kept constant (box centre coordinates x, y, z: 49.1694; 69.452; 17.6645 and box dimensions x, y, z: 26.3049, 20.3239, 28.0724). Additional docking parameters set were 10 number of binding modes, 8 exhaustiveness of search and a maximum energy difference of 3 kcal/mol.

The docking poses for each compound structure were ranked by binding affinity (kcal/mol) and RMSD values (Table S1).

Supplementary Table S4. Docking data related to ADEP7, ACP1, P4-B. P7-B, P8-B and P9-B

Ligand	Autovina Dock Score (binding affinity kcal/mol)	RMSD (l.b.)	RMSD (u.b)
**ADEP7**	-9.6	1.623	2.245
**ACP1**	-7.2	2.01	3.279
**P4-B**	-6.7	2.181	2.739
**P7-B**	-6.7	1.839	2.162
**P8-B**	-6.9	1.336	1.646
**P9-B**	-7.8	0.026	1.129

RMSD values are calculated relative to the best mode and use only movable heavy atoms. In Vina, two variants of RMSD variants are provided: RMSD lower and upper bound (l.b. and u.b., respectively), differing how the atoms are matched in the distance calculations. RSMD lower bound matches each atom in one conformation with itself in the other conformation, ignoring any

[7] M.D., Hanwell, *et al. J Cheminform* **2012**, *4*, 2-17.

[8] M.C., Bewley, *et al. J Structural Biology* **2006**, *153*, 113-128.

[9] E.F. Pettersen, et al. *J. Comput. Chem*. **2004**, *25*, 1605-1612.; O. Trott, *et al.*, *J Comput Chem.* **2010**, *31*, 455-61.

symmetry. RMSD upper bound matches each atom in one conformation with the closest atom of the same element in the other conformation. The least energetic pose and minimum RMSD values were also examined for hydrogen bonding interactions between the docked ligand and protein to confirm result validity.

Data analysis

Statistical analysis was performed by Graphpad Prism using a multiple t-test analysis. Statistical significance (P values) is defined as follows: $P > 0.05$ (ns), $P \leq 0.05$ (*), $P \leq 0.01$ (**), $P \leq 0.001$ (***).
